# Identification of microRNA clusters cooperatively acting on epithelial to mesenchymal transition in triple negative breast cancer

**DOI:** 10.1093/nar/gkz016

**Published:** 2019-01-18

**Authors:** Laura Cantini, Gloria Bertoli, Claudia Cava, Thierry Dubois, Andrei Zinovyev, Michele Caselle, Isabella Castiglioni, Emmanuel Barillot, Loredana Martignetti

**Affiliations:** 1 Institut Curie, 26 rue d’Ulm, F-75005 Paris, France; 2 PSL Research University, F-75005 Paris, France; 3 Inserm, U900, F-75005, Paris France; 4 Mines Paris Tech, F-77305 cedex Fontainebleau, France; 5 Computational Systems Biology Team, Institut de Biologie de l’Ecole Normale Supérieure, CNRS UMR8197, INSERM U1024, Ecole Normale Supérieure, Paris Sciences et Lettres Research University, 75005 Paris, France; 6 Institute of Molecular Bioimaging and Physiology, National Research Council (IBFM-CNR), Italy; 7 Institut Curie, PSL Research University, Department of Translational Research, Breast Cancer Biology Group, Paris, France; 8 Department of Physics and INFN, Università degli Studi di Torino, Turin, Italy

## Abstract

MicroRNAs play important roles in many biological processes. Their aberrant expression can have oncogenic or tumor suppressor function directly participating to carcinogenesis, malignant transformation, invasiveness and metastasis. Indeed, miRNA profiles can distinguish not only between normal and cancerous tissue but they can also successfully classify different subtypes of a particular cancer. Here, we focus on a particular class of transcripts encoding polycistronic miRNA genes that yields multiple miRNA components. We describe ‘clustered MiRNA Master Regulator Analysis (ClustMMRA)’, a fully redesigned release of the MMRA computational pipeline (MiRNA Master Regulator Analysis), developed to search for clustered miRNAs potentially driving cancer molecular subtyping. Genomically clustered miRNAs are frequently co-expressed to target different components of pro-tumorigenic signaling pathways. By applying ClustMMRA to breast cancer patient data, we identified key miRNA clusters driving the phenotype of different tumor subgroups. The pipeline was applied to two independent breast cancer datasets, providing statistically concordant results between the two analyses. We validated in cell lines the miR-199/miR-214 as a novel cluster of miRNAs promoting the triple negative breast cancer (TNBC) phenotype through its control of proliferation and EMT.

## INTRODUCTION

MicroRNAs (miRNAs) are small RNA molecules emerged as important regulators of gene expression at the post-transcriptional level. They have been shown to be involved in the regulation of all essential functions of the cells from differentiation and proliferation to apoptosis (1). Each miRNA possesses hundreds of target genes, and a single gene can be targeted by several miRNAs (2), giving rise to complex interaction networks, currently very partially characterized.

Multiple studies demonstrated the importance of miRNAs in all the cancer hallmarks defined by Hanahan and Weinberg (3) and indicated that they might function as oncogenes or tumor suppressors (4–6). Further experimental evidences suggested that specific miRNAs may also have a role beyond the cancer onset and directly participate in cancer invasiveness and metastasis (5,7). Indeed, miRNA profiles can distinguish not only between normal and cancerous tissue but they can also successfully classify different subtypes of a particular cancer (8,9), notably of breast cancer (10–12).

In this work, we focused our attention on a particular class of transcripts encoding polycistronic miRNA genes that yields multiple miRNA components. A famous example of this class of transcripts is the mir-17/92 polycistronic oncogene that plays a role in the development of various cancer types, especially in their most aggressive form (13). Genomically clustered miRNAs of mir-17/92 are simultaneously expressed and target different components of the signaling cascade as well as the downstream effectors of pro-tumorigenic signaling pathways (14–16). Deep sequencing of triple negative breast cancer (TNBC) samples revealed a threefold increase of miR-17/92 levels (11). Other studies in breast cancer have shown that mir-106b/25 cluster activates TGF-β signaling and epithelial-mesenchymal transition (EMT) (17) and miR-221/222 cluster is a key regulator of luminal breast cancer tumor progression (18).

Since >30% of annotated human miRNAs are organized in genomic clusters, we can expect to find other oncogenic/tumor suppressor polycistronic miRNAs that are co-expressed to jointly regulate molecular pathways involved in cancer malignancy. Existing computational approaches for the identification of master miRNA regulators involved in cancer onset and subtyping are typically designed to detect the effect of a single miRNA (see review in (19)). However, miRNAs have been shown to frequently act in a combined manner, jointly regulating proteins in close proximity of the protein-protein interaction network (20) and functionally related genes (21–25). The underlying assumption of this work is that this mode of action might be true also for genomically clustered miRNAs. Indeed, it has already been shown that clustered miRNAs carry out pervasive cotargeting (26).

Here, we present Clustered MiRNA Master Regulator Analysis (ClustMMRA), a fully redesigned release of the MiRNA Master Regulator Analysis (MMRA) (27,28) pipeline, developed to search for clustered miRNAs potentially driving cancer subtyping. MMRA was designed for miRNA underlying tumor subtypes, a comparison characterized by much lower variation than cancer versus normal conditions. The results of the MMRA pipeline were experimentally validated, proposing a set of four miRNAs predicted to drive the stem-like aggressive colorectal cancer subtype (27).

ClustMMRA extends MMRA to a model in which multiple miRNAs belonging to the same genomic cluster coordinately target functionally related genes driving the phenotype of a particular cancer subtype. As the MMRA pipeline, ClustMMRA is a multi-step workflow that requires in input miRNA/mRNA expression profiles from matched tumor samples classified in different subtypes according to subtype-specific gene signatures. The final output of ClustMMRA provides key miRNA clusters contributing to the regulation of particular subtypes of the disease.

We tested this novel pipeline to search for oncogenic/tumor suppressor polycistronic miRNAs driving breast cancer subtypes. ClustMMRA was applied to two independent breast cancer datasets whose samples were previously classified into four subtypes (luminal A, luminal B, HER2+ and triple negative). We obtained statistically concordant results between the two analyses, identifying five clusters of miRNAs with aberrant expression in a specific subtype of both datasets. Among them, miR-199a/214 on chromosome 1 was found to be down-regulated in the triple negative subtype and associated to EMT regulation. Functional validation in cell lines confirms the regulatory effect of this cluster in shaping the triple negative subtype phenotype through its control of proliferation and EMT. Overall, our computational pipeline and experimental validations characterize a new genomic cluster of miRNAs implicated in the TNBC phenotype that might be further explored in diagnosis and therapeutic strategies. In addition, we evinced a cooperative mechanism for the regulatory activity of genomically clustered miRNAs.

## MATERIALS AND METHODS

### MiRNA cluster annotation

The genomic locations of miRNAs were retrieved from miRBase v18 (29). As in (30,31), co-clustered miRNAs are defined as *miRNA* genes located within 10 kb of distance on the same chromosome and in the same strand.

### Datasets preprocessing

Breast cancer (BRCA) RNA-seq and miRNA-seq Level 3 expression profiles were downloaded from The Cancer Genome Atlas (TCGA) in January 2016. Only those primary tumors profiled for both mRNA and miRNA expression were included in the analysis, obtaining a total of 397 samples. Two expression matrices (one for mRNAs and the second for miRNAs) were normalized obtaining the paired mRNA/miRNA expression dataset here referred to as TCGA. The Curie dataset was generated with microarray technologies (Agilent miRNA microarray kit V3 for miRNAs and Affymetrix U133plus2 for mRNA) and pre-processed following the procedure described in (32). Experiments were conducted in agreement with the Bioethic Law No. 2004–800 and the Ethic Charter from the French National Institute of Cancer (INCa), and after approval of the ethics committee of our Institution. Finally the paired microRNA-mRNA expression dataset of colorectal cancer was downloaded from (27).

### Definition of a gene signature for each breast cancer subtype

The ClustMMRA pipeline requires as input a gene signature for each disease subtype. Available signatures for breast cancer subtypes, such as the PAM50 (33), were not applicable here due to their limited size in terms of number of genes. We thus defined the signatures for our breast cancer study using the approach proposed in (34). The Curie dataset was used for signature construction, while the TCGA dataset was employed for signature validation. Differential gene expression for each subtype vs. all the other samples was computed by Student's *t*-test and log fold change cutoff (*t*-test adjusted *P*-value < 0.05 and absolute(log fold change) > 0.5). Moreover, to increase the predictive power of the constructed signatures, those genes associated to more than one class according to the previous criteria, or having a difference between the first and second highest absolute(log fold changes) lower than 0.2 were discarded. The choice of thresholds was optimized to maximize the gene association to a unique subtype and the number of genes included in each signature (on average 117 genes per signature). For each subtype, two separated signatures were defined (‘down' and ‘up'), based on the sign of the expression change of their genes. The .gmt file of the constructed signatures is available at https://github.com/lcan88/Supplementary_miRNA_cluster/blob/master/signature_Breast.gmt. The reliability of these signatures were tested in two ways. First, their classification performances were validated on TCGA data. We classified the TCGA samples using our signatures with the Nearest Template Prediction (NTP) method (35), as done in (36,37). Only 44 out of 397 (11%) samples resulted to be misclassified. Then, the significance of the intersection between our signatures and publicly available ones was evaluated by a Fisher's exact test. The signatures used for this test were obtained from MSigDB (38) plus a specific one derived from (39). The proliferation signatures were added to test the TNBC subtype, known to be associated to a strong proliferative signal. Highly significant *P*-values were obtained for the intersection between our newly defined signatures and previously published ones for the same breast cancer subtypes. The above results confirm the classification performances and reliability of the breast cancer signatures here constructed.

### From single miRNA to clusters of miRNAs: ClustMMRA

**Figure 1.**
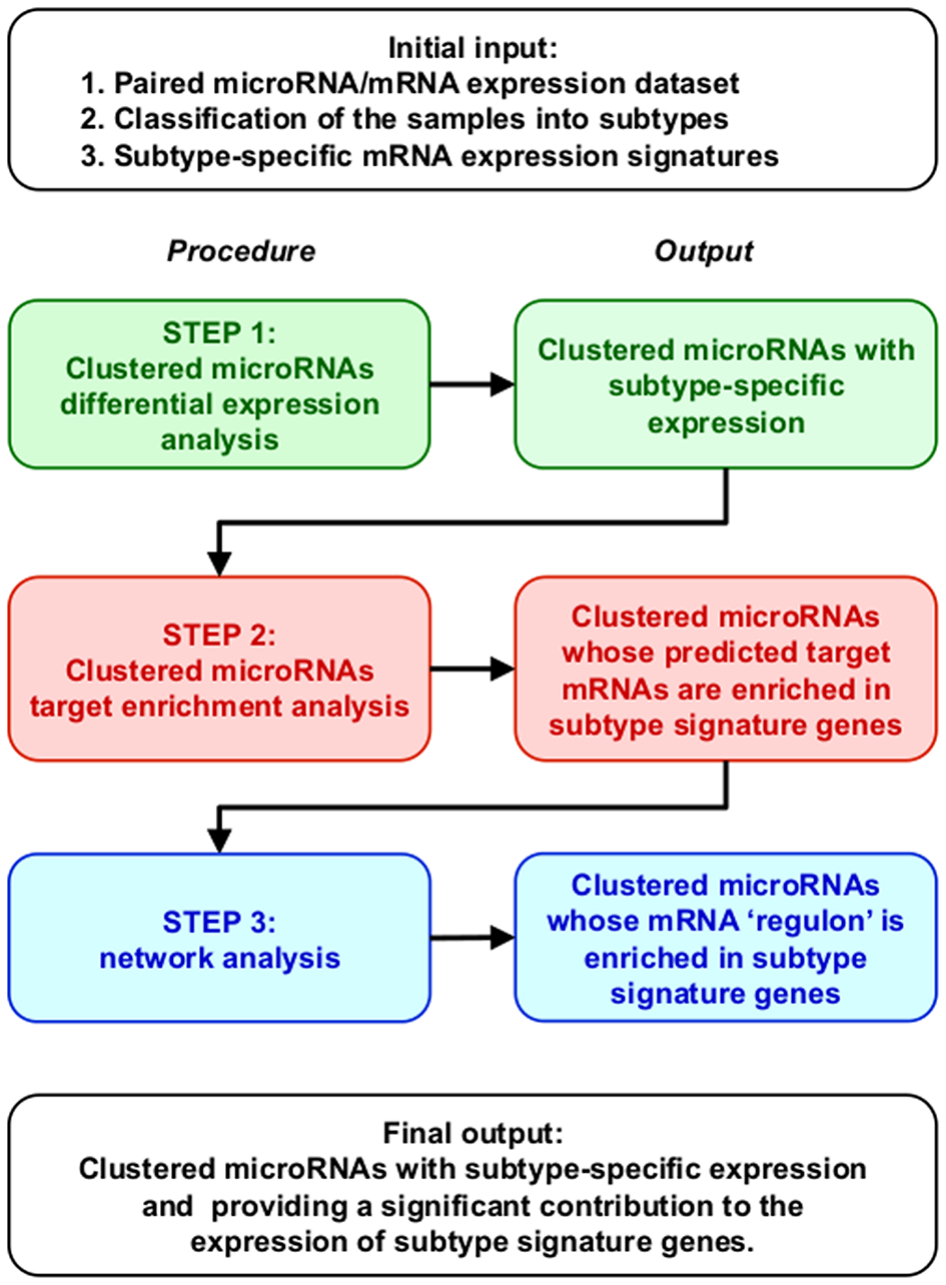
Schematic representation of the Clustered microRNA Master Regulator Analysis (ClustMMRA) workflow. The schema reports the data required as initial input, the four analytical steps with the respective outputs, and the final output of the pipeline.

The MMRA pipeline is here extended to search for genomically co-clustered miRNAs potentially driving cancer subtyping. Similar to MMRA, the workflow of ClustMMRA (see Figure 1) consists of subsequent filtering steps: (i) differential expression analysis of clustered miRNAs; (ii) target enrichment analysis and (iii) network analysis. While a miRNA cluster is usually transcribed as a single unit (40–44), the expression of mature miRNAs in the same cluster might not be highly correlated due to regulatory events in the maturation processes (40,43). ClustMMRA is available at https://github.com/lcan88/clustMMRA.

Clusters of miRNAs are identified based on their genomic organization as reported in Methods. In step (i), the subtype-specific expression of each miRNA is assessed by Kolmogorov–Smirnov (KS) statistical test and fold change cutoff. Clusters having at least two miRNAs with subtype-specific expression change in the same direction (both up-regulated or down-regulated) are selected for step (ii).

In step (ii), we extract miRNA clusters having their predicted targets enriched for the gene signature of the corresponding subtype. Only miRNAs of the cluster classified as differentially expressed in step (i) are considered in step (ii). The targets of individual miRNAs have been predicted using four different databases (TargetScan 7.1, doRiNA-PicTar 2012, microRNA.org 2010, PITA 2007) plus an experimental one (miRTarBase 2.5) and requiring the prediction by at least two of them. Changes in the results of the pipeline when the correlation between the expression profiles of the predicted miRNA-target interactions is also used are reported in Supplementary Text. The set of targets of a cluster has been defined as the union of the targets of individual miRNAs. To control the number of false positive results we defined a threshold for the significance of the overlap between the targets of the cluster and the genes present in the signature based on a null model. For 1000 times random set of genes of the same size of the set of targets are generated and tested for their intersection with the gene signature. The 95th percentile of the obtained distribution is used as threshold. The objective of step (i) and (ii) is to identify co-clustered and co-expressed miRNAs potentially regulating a gene expression signature in a joint manner, without necessarily having a high overlap in terms of target genes (22). Finally, in step (iii) a miRNA–mRNA interaction network is constructed for each selected cluster using the ARACNE algorithm (45,46) and employing all the expressed genes. In this step, we identify modules of co-clustered miRNAs and interacting genes, including indirect interactions, that are believed to participate in the phenotype of a given cancer subtype (we call these modules *regulons*). Unlike the results of the MMRA pipeline, in which *regulons* can include only one miRNA, the ones identified by the ClustMMRA pipeline contain multiple miRNAs of the genomic cluster. Interference of indirect interactions may introduce links between miRNAs and spurious genes in the *regulon*. A Fisher's exact test has been performed to evaluate the statistical significance of the overlap between the genes included in each *regulon* and the gene signature of the associated subtype.

### Cell culture and miRNA modulation

For *in vitro* studies, we used two human BC epithelial cell lines: T47D and MDA-MB-231 cells (ICLC-Biologic Bank and Cell Factory, Italy). These cell lines were chosen as they represent a model of luminal A and TNBC cell lines, respectively (47). Following the manufacturer's recommendation, we maintained the cell lines within a humidified atmosphere containing 5% CO_2_ at 37°C in DMEM (for T47D cell line) or advanced DMEM (for MDA-MB-231 cell line) cell culture medium (Gibco, Life Technologies), with 10% fetal bovine serum (FBS), 1% penicillin-streptomycin, 2 mM glutamine (all from Lonza, Euroclone). Dulbecco phosphate-buffered saline (D-PBS), trypsin, and all the media additives were obtained by Lonza (Euroclone).

The sense (S) oligonucleotide sequence of each miRNA of the cluster has been designed following the sequences indicated in miRbase database (29). S oligonucleotides were purchased from Sigma.

To obtain the upregulation of each miRNA, S oligonucleotides, resuspended in water, were added three times a day for 3 days directly to the culture medium of the cells (<50% confluency) at a final concentration of 100 nM/day (48). The cells were collected 24, 48 or 72 h of treatment and different assays were performed (proliferation, real time-PCR analysis of miRNAs and EMT genes and ECM assay).

### Proliferation assay

Tumor cell proliferation was assessed by following the protocol described in (49). Briefly, cells were seeded at a confluency of 80 000 cells/w in 24-well plates. The cells were added daily with 100 nM final concentration of S miR-214, -199a-3p, -199a-5p. The proliferation assay was performed either counting the cells at 24, 48 and 72 h of treatment or by staining the alive cells with thiazolyl blue tetrazolium bromide (MTT, SIgma Aldrich), following supplier's suggestions. The cell absorbance was counted at 540 nm at indicated time points. A graphic representation of the counts was obtained by plotting the average value of Absorbance at each time point of three independent experiments, performed in triplicate (*n* = 9).

### ECM assay

For extracellular matrix (ECM) staining, cells were grown for 3 days before fixation with 100% cold methanol at –20°C (Sigma). Then, cells were permeabilised with 0.1% Triton X-100 (Euroclone, Italy) in PBS 1× before staining with anti-collagen I (Aurogene, Italy; 1:100). Anti rabbit 488 (AlexaFluor, Life Technologies) secondary antibody was used for the staining. Images were acquired using a Leica DMIL microscope (4×, 10×, 20×, 40× objectives) with Leica ICC50W camera. Images were quantified with ‘corrected total cell fluorescence (CTCF) method’, where the integrated density was subtracted with the area of selected cells multiplied for the mean fluorescence of the background readings (50,51).

### RNA isolation, reverse transcription and RT-PCR analysis

Total RNA was isolated using TRIzol reagent (Life Technologies) following the manufacturer's recommendations. To obtain cDNA from total RNA for gene expression analysis, two micrograms of total RNA were reverse transcribed using oligo dT primers in combination with High Capacity cDNA Reverse Transcription kit (Applied Biosystem), following the manufacturer's protocol.

For miRNA analysis, one microgram of total RNA was reverse transcribed using MystiCq microRNA cDNA synthesis kit (Sigma), following the manufacturer's protocol, in order to reverse transcribe polyA-tailed miRNA into cDNA.

RT-PCR analysis was performed using Power Up Sybr Green Master mix (Applied Biosystem, Life Technologies) in an Eco RT-PCR machine (Illumina). All the primers for human mRNA and miRNA amplification were home-made and are described below (Supplementary Table S1). miRNA amplification was performed using primers designed on the mature miRNA sequence taken from miRbase v18.

The relative expression of miRNAs and genes was calculated for both T47D and MDA-MB-231 cell lines with the 2^(−ΔΔC^_T_^)^ method (52). Experiments were performed three times in triplicate (*n* = 9). A *t* test was calculated.

## RESULTS

### Identification of regulatory miRNA clusters underlying breast cancer subtypes

We applied ClustMMRA (https://github.com/lcan88/clustMMRA) to identify polycistronic miRNAs underlying breast cancer molecular subtypes. For this study, two independent datasets were used, a first paired miRNA/mRNA expression dataset from a in-house cohort of 129 breast carcinoma tumour samples (which we refer to as Curie dataset (53,54) and a second dataset from The Cancer Genome Atlas project composed of 397 samples (55). In both datasets, individual samples were assigned to four subtypes (luminal A, luminal B, HER2+ and triple negative) based on the immunohistochemical staining of estrogen (ER), progesterone (PR) and HER-2 (ERBB2) receptors.

### ClustMMRA application to Curie and TCGA datasets

Expression data required for running ClustMMRA were pre-processed as described in Methods and the signatures for breast cancer subtypes were defined using the approach proposed in (34) (see Methods). We applied the ClustMMRA pipeline on Curie and TCGA datasets separately. In the first step, genomically co-clustered miRNAs having a subtype-specific expression were identified. In this step, 28 and 47 out of 131 analyzed clustered miRNAs were selected for Curie and TCGA datasets, respectively (see Supplementary Table S2). Of these, 18 clusters were in common between the two datasets (*P*-value < 7e–04), revealing a significantly concordant expression pattern of co-clustered miRNAs. Among these co-clustered and co-expressed miRNAs, some are differentially expressed in multiple subtypes (18 and 37 clusters for Curie and TCGA respectively), with 15 out of 18 and 21 out of 37 differentially expressed in TNBC and luminal A with opposite sign.

In step (ii), 10 out of 28 (Curie) and 16 out of 47 (TCGA) subtype-specific miRNA clusters were found to have their predicted targets enriched in genes belonging to the corresponding gene signature. The output of step (ii) (see Supplementary Table S3) has an intersection of 7 elements between the two datasets (*P*-value < 1e–05). In the step (iii) of ClustMMRA, a *regulon* for each miRNA cluster selected in step (ii) was constructed. As expected, in the constructed regulons, the miRNA targets tend to have a significantly higher Mutual Information with the miRNA in respect to the other non-target genes (see Supplementary Figure S1). The *regulons* were tested for enrichment in gene signature. Seven out of 10 and 9 out of 16 clusters passed this last selection step in Curie and TCGA datasets, respectively. These clusters constitute the final output of ClustMMRA and are reported in Table 1. After this last step, the output in common between the two datasets contains five clusters (*P*-value < 8e–06). The significant overlap between results obtained from the analysis of two independent datasets with ClustMMRA supports the high reproducibility across independent datasets of this approach. Notably, the results have an intersection with increasing statistical significance at each step of the pipeline. This trend confirms the accuracy of the proposed pipeline in selecting candidate clusters underlying cancer subtypes.

**Table 1.** Clusters of miRNAs identified by ClustMMRA in breast cancer TCGA and/or Curie datasets

Cluster of miRNAs	Chromosome position	Number of deregulated miRNAs in the cluster	Cluster expression in subtypes	Gene signature expression in subtypes	Dataset results	Also in CRC
**miR-199a/214**	Chr1	3	Down in TNBC	Up in TNBC	**Curie and TCGA**	**no**
**miR-493/136**	Chr14	8	Down in TNBC	Up in TNBC	**Curie and TCGA**	**no**
**miR-379/656**	Chr14	42	Down in TNBC	Up in TNBC	**Curie and TCGA**	**yes**
**miR-512/373**	Chr19	46	Up in TNBC	Up in TNBC	**Curie and TCGA**	**yes**
**miR-532/502**	ChrX	8	Up in TNBC	Down in TNBC	**Curie and TCGA**	**no**
miR-449a/449c	Chr5	3	Down in TNBC	Down in TNBC	TCGA	no
miR-653/489	Chr7	2	Down in TNBC	Down in TNBC	TCGA	yes
miR-548aa/548d	Chr8	2	Up in TNBC	Down in TNBC	TCGA	no
miR-421/374c	ChrX	3	Up in TNBC	Up TNBC	TCGA	yes
miR-99a/let-7c	Chr21	2	Down in TNBC	Up TNBC	Curie	no
miR-450b/424	ChrX	6	Down in TNBC	Up TNBC	Curie	yes

Some results obtained with ClustMMRA in the breast cancer study have already been validated in the literature. MiR-493/136 and miR-379/656 clusters in the chromosomal region 14q32 have been reported as tumor suppressors in different types of human cancer (56–58), including breast cancer (59). Silencing of multiple miRNAs encoded in these clusters was shown to increase the proliferation and invasion of ovarian (60), melanoma (61) or oral squamous carcinoma (56) cells. The X-chromosome-located miR-532/502 cluster has been previously associated to cancer. In particular, this was found up-regulated in triple-negative breast cancer cells (62) and the regulatory circuit miR-502/H4K20 methyltransferase SET8 was described as a key regulator of breast cancer pathobiology (63).

### Half of the identified miRNA clusters are cancer-type specific

Multiple studies have identified miRNAs associated with tumor development and progression in most major cancer types (64–66). To test whether some of the clusters identified by clustMMRA in breast cancer have a driving role also in other cancer types we repeated the analysis in Colorectal Cancer (CRC). We considered in particular the paired microRNA-MRNA expression dataset of 450 samples used in (27). As in (27), the samples were partitioned into subtypes according to the CRCA classifier (34). At step (i) 19 clusters were found to be differentially expressed across the CRCA subtypes (see Supplementary Table S4). Of them, 18 were also found to have a significant number of targets in the corresponding gene signatures (see Supplementary Table S5). Finally, nine clusters constituted the output of clustMMRA in CRC (see Supplementary Table S6).

We then tested the overlap between the output of ClustMMRA in CRC and in breast cancer. Approximately 50% of the clusters output of clustMMRA in breast are also present in CRC (four out of nine in TCGA and three out of seven in Curie) indicating that many of the identified clusters of miRNAs have a regulatory role across multiple cancer types.

### Comparison of ClustMMRA with the pipeline for the identification of single master miRNA regulators (MMRA)

We compared the results of ClustMMRA in breast and colorectal cancer with those obtained by applying to the same datasets the MMRA pipeline for the identification of single master miRNA regulators. The goal is to investigate if the regulatory effect of a cluster can be detected by studying the effect of individual miRNAs belonging to the same cluster.

We applied MMRA to the Curie and colorectal cancer (TCGA) datasets, using in each step the same thresholds employed for ClustMMRA. If at least two miRNAs of a given cluster are included in the output of MMRA, we consider this cluster as detected in the single-miRNA pipeline. Interestingly, four out of seven clusters detected by ClustMMRA (miR-199a/214, miR-493/136, miR-512/373 and miR-450b/424) were not detected by MMRA in Curie. Concerning CRC, seven out of nine miRNA clusters detected by clustMMRA are lost in MMRA.

This difference between the outputs of the two pipelines is given by the target enrichment analysis in step (ii) and the network analysis in step (iii). In fact, the four clusters missing in the final output of MMRA in Curie are included in the output of step (i), since they have at least two differentially expressed miRNA genes. They are filtered out in step (ii) since no miRNA gene in these clusters, when analyzed individually, reaches a significant enrichment of signatures genes in its targets for a certain subtype. This observation supports the hypothesis that co-clustered miRNAs participate in regulating the gene expression signature of a given cancer subtype without necessarily having a high overlap in terms of common target genes.

### Prioritization of miRNA clusters for functional validation in cell lines

Before experimental validation of the ClustMMRA output, prioritization of results was performed. We considered the five clusters identified both in TCGA and Curie datasets. For the *regulons* associated to each cluster, the nodes present in both TCGA and Curie datasets were kept, obtaining a network for each *regulon* with size of about 100 nodes. Then, biological processes and pathways associated to these *regulons* were identified through Fisher's exact enrichment test, using MSigDB (38) as reference collection of signatures for pathways and biological functions. The complete list of MSigDB pathways resulting from this analysis (FDR < 0.05) is reported in Supplementary Table S7.

**Figure 2.**
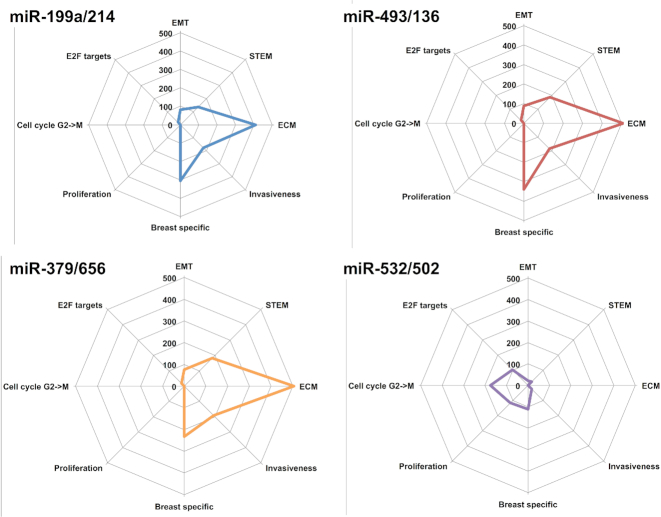
Pathways controlled by the deregulated miRNA clusters. A summary of the main biological functions controlled by the different miRNA clusters is here reported. Y-axis of the radarplot corresponds to the sum of the absolute log(*P*-value) of all the pathways associated to a given function.

As summarized in Figure 2, the network analysis shows a regulation of EMT, stemness and extracellular matrix by clusters miR-493/136, miR-379/656 and miR-199a/214. Cluster miR-532/502 is predicted to regulate proliferation and the cell cycle transition from G to M phases. All the *regulons* have been found associated to breast cancer specific signatures, with clusters miR-493/136, miR-379/656 and miR-199a/214 sharing nine of them (‘SCHUETZ_BREAST_CANCER_DUCTAL_INVASIVE_UP’,‘FARMER_BREAST_CANCER_CLUSTER_4’,‘TURASHVILI_BREAST_LOBULAR_CARCINOMA_VS_LOBULAR_NORMAL_DN’,‘CHARAFE_BREAST_CANCER_LUMINAL_VS_MESENCHYMAL_DN’,‘LANDIS_BREAST_CANCER_PROGRESSION_DN’,‘LANDIS_ERBB2_BREAST_TUMORS_324_DN’,‘LIEN_BREAST_CARCINOMA_METAPLASTIC’,‘TURASHVILI_BREAST_DUCTAL_CARCINOMA_VS_DUCTAL_NORMAL_UP’,‘TURASHVILI_BREAST_LOBULAR_CARCINOMA_VS_DUCTAL_NORMAL_UP’,‘TURASHVILI_BREAST_LOBULAR_CARCINOMA_VS_LOBULAR_NORMAL_DN’). Invasive and mesenchymal state signatures confirm the association of these clusters to the TNBC subtype. Other general processes were found enriched in the regulons of these clusters: EMT (including the ‘HALLMARK_EPITHELIAL_MESENCHYMAL_TRANSITION’ signature and multiple GO terms related to the extracellular matrix), stemness (‘BOQUEST_STEM_CELL_UP’,‘LIM_MAMMARY_STEM_CELL_UP’,‘IZADPANAH_STEM_CELL_ADIPOSE_VS_BONE_DN’ signatures), cell cycle (‘IGLESIAS_E2F_TARGETS_UP’) and angiogenesis (‘GO_VASCULATURE_DEVELOPMENT’,‘GO_CIRCULATORY_SYSTEM_DEVELOPMENT’). Finally, the regulon of cluster miR-532/502 was found enriched in some breast cancer specific signatures clearly linking it to the TNBC subtype (‘SOTIRIOU_BREAST_CANCER_GRADE_1_VS_3_UP’,‘FARMER_BREAST_CANCER_BASAL_VS_LULMINAL’ and ‘POOLA_INVASIVE_BREAST_CANCER_UP’). Also, it was observed to be strongly associated to proliferation signatures (e.g. ‘ZHOU_CELL_CYCLE_GENES_IN_IR_RESPONSE_24HR’,‘GO_MITOTIC_NUCLEAR_DIVISION’,‘GO_MITOTIC_CELL_CYCLE’,‘GO_CHROMOSOME_SEGREGATION’,‘GO_CELL_DIVISION’,‘GO_CELL_CYCLE_PROCESS’,‘CHANG_CYCLING_GENES’).

### Functional validation of miR-199a/miR-214 cluster in breast cancer cell lines

To functionally validate our *in silico* results we considered miR-199a/miR-214 cluster, predicted to regulate TNBC according to clustMMRA. Considering the technical difficulty in producing the over-expression of multiple miRNAs in cell lines, we chose this cluster being the smallest of our output. Given that miR-199a/miR-214 is underexpressed in TNBC, its overexpression in a TNBC cell line should make the cell shift away from the TNBC subtype. We used as a measure to evaluate such a shift proliferation, in fact TNBCs are known to have a higher proliferation index than the other breast cancer subtypes (67).

#### MiR-199a/miR-214 cluster is underexpressed in TNBC cells

Human miR-199a/miR-214 cluster is encoded by a large non-coding RNA on chromosome 1q24 which produces three mature miRNAs (hsa-miR-199a-5p, hsa-miR-199a-3p and hsa-miR-214). First, we examined by quantitative RT-PCR the expression of the individual mature miRNAs belonging to this cluster in T47D and MDA-MB-231 cells, which are luminal A and TNBC cells respectively (47). Results show that the three mature miRNAs encoded by the miR-199a/miR-214 cluster are significantly underexpressed in MDA-MB-231 compared to T47D cells (Supplementary Figure S2).

#### Upregulation of miR-199a/miR-214 cluster decreases TNBC cell proliferation

**Figure 3.**
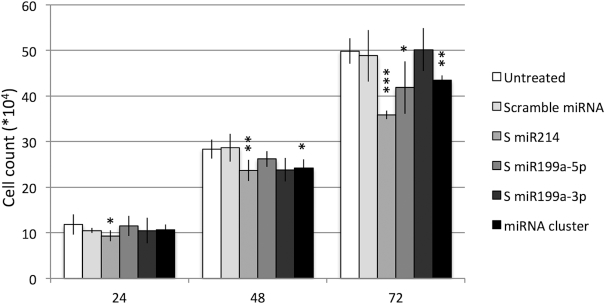
In vitro analysis of miRNA modulation effect on MDA-MB-231 cells proliferation. MDA-MB-231 cells were treated for 24,48,72 hours (h) with sense (S) oligonucleotide encoding for miRNA cluster or single miRNA (miR-214, miR-199a-5p, miR-199a-3p) or a scramble miRNA. The effect of miRNA modulation on cell proliferation is shown. Average±sd of three independent experiments for each cell line are shown. *t*-test *P*-value < 0.001 (***), <0.01 (**), <0.05 (*).

To test whether the deregulation of miR-199a/miR-214 cluster was sufficient to impact TNBC cells phenotype, MDA-MB-231 cells were treated with sense (S) oligonucleotides encoding for all the three miRNAs of the cluster (miR-214, miR-199a-5p, miR-199-3p) or scramble negative controls. We checked the overexpression of each miRNA of the cluster after transfection by RT-PCR analysis, shown in Supplementary Figure S3. After confirming the upregulation of single miRNA or all three miRNAs of the cluster in MDA-MB-231, we analyzed the effect of miRNA overexpression on proliferation: individual miRNAs, except miR-199a-3p, and entire miR-199a/miR-214 cluster overexpression reduce the MDA-MB-231 cell number compared to scramble or untreated control (Figure 3).

We then focused on the regulation of EMT predicted by clustMMRA (see Figure 2) as an interesting phenotype to validate in TNBC subtype.

#### MiR-199a/miR-214 cluster silencing is associated with EMT-like and invasive phenotype

**Figure 4.**
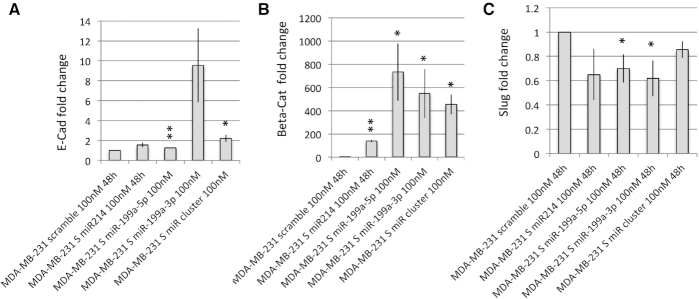
Effect of miRNA modulation on EMT marker genes. MiRNA modulated MDA-MB-231 cells were used for RT-PCR analysis of EMT marker genes. RT-PCR analysis shows the effect of single miRNA or miRNA cluster modulation vs scramble oligonucleotide treated cells on E-cadherin (**A**), beta-catenin (**B**) and slug (**C**). Average±sd of three independent experiments for each cell line are shown. *t*-test *P*-value <0.01 (**), <0.05 (*).

According to our results, miR-199a/miR-214 cluster is also predicted to modulate EMT genes and cell invasion. To investigate if the expression of this cluster affects the molecular profile of the cells, we analyzed the expression levels of EMT-related genes upon upregulation of a single miRNA of the cluster or the whole cluster through S oligonucleotide treatment. We observed a reduction of EMT marker genes upon both individual miRNAs or entire miR-199a/miR-214 cluster overexpression (Figure 4), as demonstrated by the increase expression of epithelial markers E-cadherin and Beta-catenin and a decrease of the expression level of the mesenchymal marker Slug.

As a negative control we repeated the same experiment on miR-502/532 cluster also associated by clustMMRA to the TNBC subtype. This cluster was predicted to control proliferation, but not EMT (see Figure 2). As shown in Supplementary Figures S4 and S5 our experiments confirm the predictions of clustMMRA.

**Figure 5.**
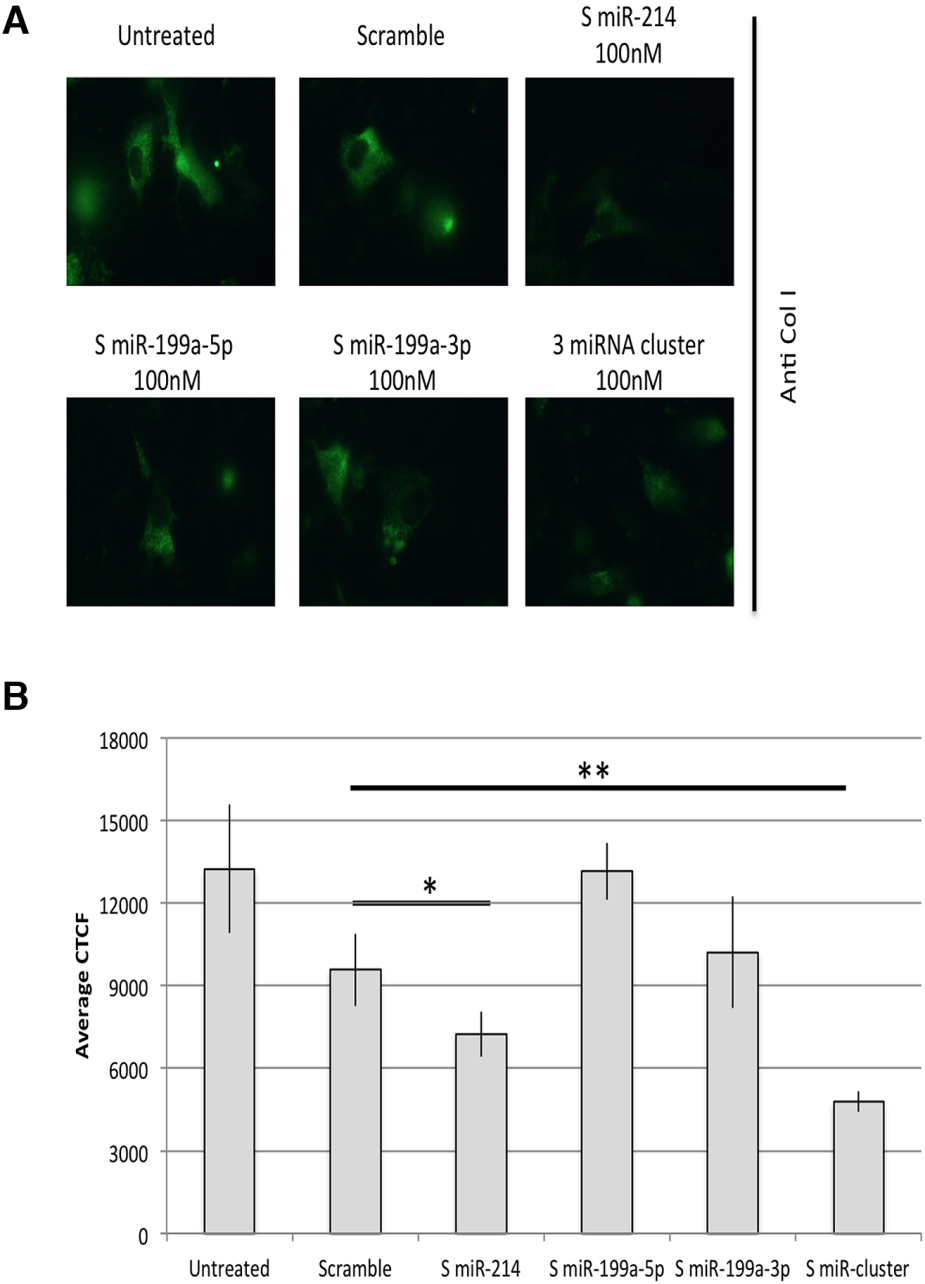
Extracellular matrix (ECM) immunofluorescence assay: collagen I staining. Average of the values of fluorescence intensity of Collagen I (Col I) of single cells, after background correction. Col I immunofluorescence was performed and pictures were taken at the same exposure (1 s) and gain (6.8). The more representative pictures for each sample are shown (**A**). Single picture fluorescence intensity was quantified by ImageJ analysis software (*n* = 6 images for each sample), and CTCF method was applied and quantified (**B**) (*t* test *n* = 6, *P* value < 0.05, *; *P* value < 0.01, **).

Finally, to further confirm the regulation of EMT by miR-199a/miR-214 cluster, we evaluated its modulatory effect on the Extracellular Matrix (ECM). We thus performed an ECM assay in cells overexpressing miR-214, miR-199a-5p, miR-199a-3p alone or the cluster. This assay consists in the analysis of the effect of miRNA overexpression on ECM deposition by immunofluorescence of one component of the extracellular matrix (in our case collagen 1, Col1). Some representative pictures for each sample are shown in Figure 5A. In Figure 5B we quantified the signal emitted by the immunofluorescence using the CTCF method described in (50,51). Both miR-214 alone or in combination with miR-199a within the cluster decreased significantly the deposition of ECM protein components (Col1), suggesting that the components of the cluster specifically control the deposition of the proteins of the ECM.

## DISCUSSION

Over the last two decades there has been an explosion of research focused on miRNAs involvement in cancer initiation and progression, pointing out the potential of these small RNAs as biomarkers for diagnosis, prognosis and response to treatment. However, the majority of computational and experimental approaches for the identification of master miRNA regulators involved in cancer onset and subtyping are typically designed to detect the regulatory effect of a single miRNA. This can be a limitation in identifying regulation by multiple miRNA species acting cooperatively on cellular pathways and pathological changes.

The computational pipeline here described, ClustMMRA, was specifically designed to search for genomically clustered miRNAs potentially driving cancer subtyping. ClustMMRA provides a computational framework to systematically investigate polycistronic miRNA transcripts involved in cancer subtyping or possibly in other biological contexts. In practice, the use of ClustMMRA can be generalized in order to study other classes of cooperatively acting miRNAs than the case of genomic clusters, such as co-expressed miRNAs from different genomic locations.

In our study, ClustMMRA was applied to search for oncogenic / tumour suppressor polycistronic miRNAs driving breast cancer subtypes, pointing out five novel miRNA clusters whose regulatory effect is potentially associated to the triple negative subtype phenotype. Among them, the miR-199/miR-214 is identified as acting on EMT in TNBC subtype. Our computational and experimental validation of the regulatory effect of miR-199/miR-214 show that the down-regulation of this genomic cluster is associated to appearance of EMT-like phenotype in the TNBC cells. The upregulation of individual miRNAs belonging to the cluster or the entire cluster decreases the expression of a marker of mesenchymal phenotype (i.e. Slug) and increases the expression of epithelial markers (E-cadherin and beta-catenin). The effects on EMT obtained by the overexpression of miR-199/miR-214 cluster are further confirmed by its control on the deposition of the ECM proteins. Our results suggest that this cluster of miRNAs is possibly involved in the maintenance of more aggressive phenotypes of breast cancer, by regulating EMT target genes, and cell proliferation. Finally, our study supports the hypothesis of miRNA cooperativity from a polycistronic transcript as a possible mechanism of joint targeting to act on molecular pathways involved in cancer malignancy and subtyping. More accurate measurements and quantitative study might improve the understanding of these cooperative effects.

## Supplementary Material

Supplementary Data
