# Supplementary material for: Identification of microRNA clusters cooperatively acting on epithelial to mesenchymal transition in triple negative breast cancer
Source: Nucleic Acids Res. 2019 Jan 18;47(5):2205–15. doi: 10.1093/nar/gkz016 (PMC6412120; doi:10.1093/nar/gkz016)
Supplement: Supplementary Data [file gkz016_supplemental_files.pdf]

Supplementary Text

Effects of the use of correlation in the step (ii) of clustMMRA

The step (ii) of clustMMRA employs miRNA-target interaction obtained from experimental and predictive databases without verifying if such interactions hold true in the biological context under study (or equivalently, we do not use the expression data to validate the miRNA-target interactions). This choice is aimed at maintaining step (ii) independent from the following one based on network reconstruction using expression profiles. Indeed, if some miRNA-target interactions identified at step (ii) do not hold true in the tissue under analysis, then in the network reconstructed through ARACNE we should not find any evidence of regulation of the miRNA cluster on the associated gene signature and thus the miRNA cluster should not be present in our output.

To further test this point, we analyzed the results of a clustMMRA variant that only considers as miRNA targets those genes present in at least 2 predictive databases and whose expression data have a significant correlation with the miRNA. In detail, once a miRNA cluster X has been associated to the cancer subtype Y according to the step (i) of clustMMRA, in the step (ii) of the tested variant of clustMMRA, we check if the set of targets of X is enriched for the gene signature of subtype Y. For each miRNA contained in X, we consider as its targets the genes predicted by at least 2 of the 5 databases (TargetScan 7.1, doRiNA-PicTar 2012, microRNA.org 2010, PITA 2007 and miRTarBase 2.5) whose expression is also significantly correlated/anticorrelated with the expression of the miRNA (pvalue lower than 0.05). We then consider as targets of cluster X, the union of all the genes target of at least one of the miRNA in cluster X. We construct a null model to define the threshold of significant intersection between the targets of X and the gene signature of Y. To do this, we consider 1000 random sets of genes of the same size of the targets of X and we test the intersection of the random sets with the gene signature of Y. The 95th percentile of the distribution defined in the null model is used as threshold.

Such variant of clustMMRA identified a lower number of clusters compared to the original pipeline: 3 clusters (miR-493/136, miR-449a/449c and miR-421/374c) out of 9 in the TCGA dataset and 2 clusters (miR-493/136, miR-99a/let-7c) out of 7 in the Curie dataset. Of note, two clusters included in the original results and already validated in literature, miR-379/656 and miR-532/502, are discarded by the variant of clustMMRA. Moreover, the cluster mir-214/199a that has been functionally validated in our study would not have been identified imposing the correlation. Also using a less restrictive one (p-value < 0.1) we would not have identified mir-214/199a in our output. These results suggest that the enrichment for signature genes of a tumor subtype in the targets predicted for a miRNA cluster can be lost if both sequence-based prediction and significant correlation between the expression profiles are required to define miRNA targets.

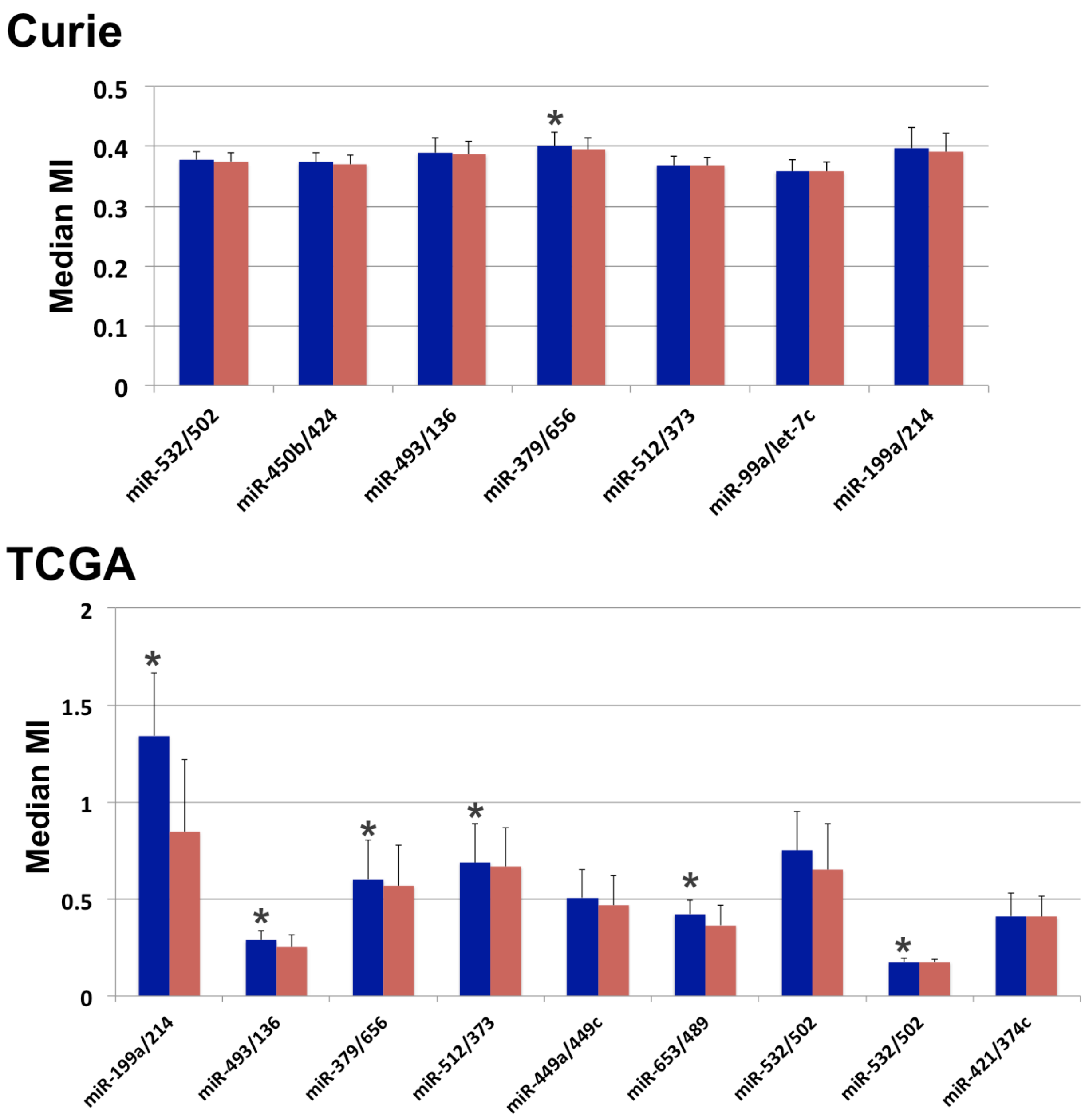

Supplementary Figure 1. Average Mutual Information (MI) values for the target (blue) and non-target (red) genes in the regulons of the miRNA clusters output of clustMMRA. Significant differences (P-value lower than 0.05) are denoted with *.

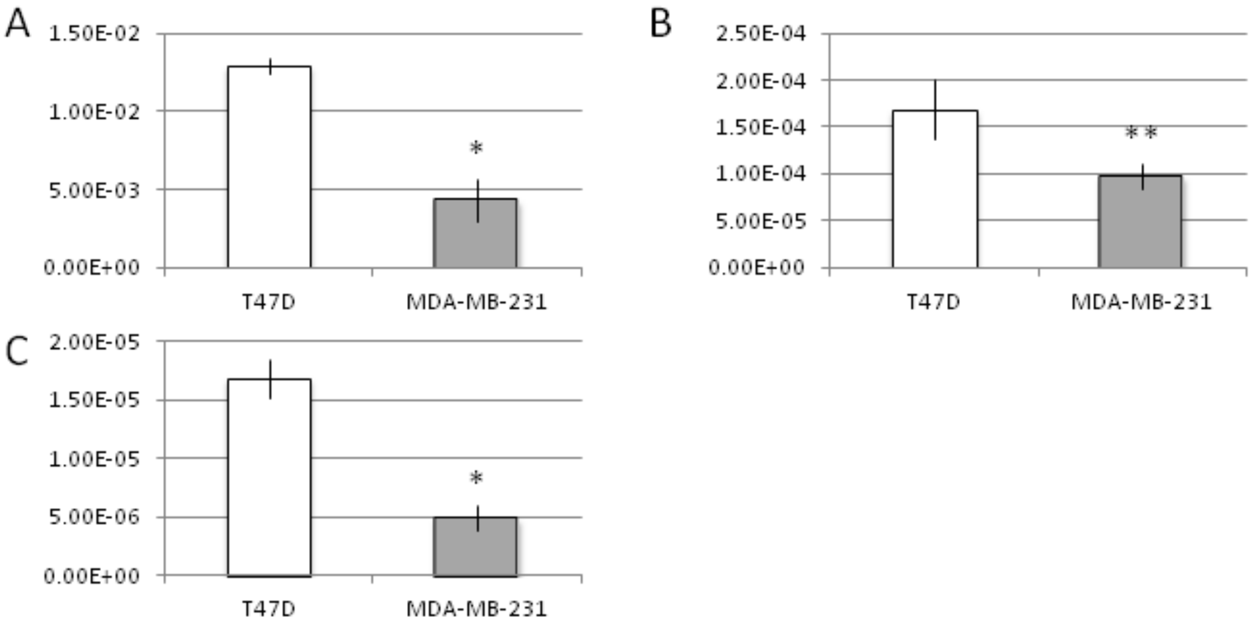

Supplementary Figure 2. RT-PCR analysis of miRNA expression in T47D vs MDA-MB-231.

T47D (in white) and MDA-MB-231 (in grey) were analyzed for the expression of miR-214 (A, p-value<0.011), miR-199a-5p (B, p-value<0.003) and miR-199a-3p (C, p-value<0.03). 2^-DDCt method was used for evaluating the expression level of each miRNA. Average±sd of three independent experiments for each cell line are shown. T-test p-value<0.01(**), <0.05(*).

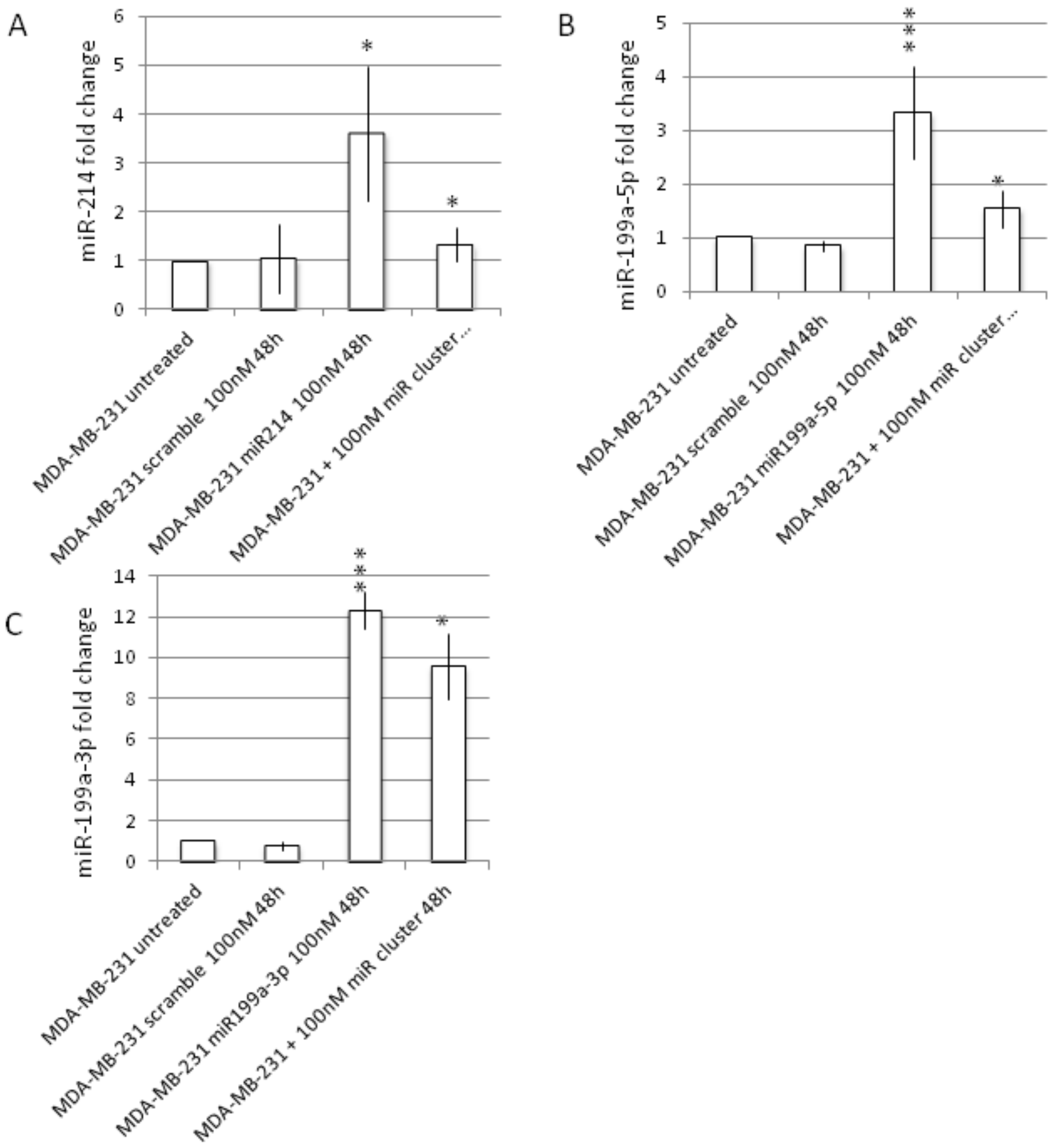

Supplementary Figure 3. Mirna modulation in MDA-MB-231 cells.

MDA-MB-231 cells were treated for 48 hours with 100nM sense (S) oligonucleotide encoding for miR-214, miR-199a-5p, miR-199a-3p or miRNA cluster, respectively. The expression levels of miR-214 (A), miR-199a-5p (B) and miR-199a-3p (C) were evaluated by RT-PCR analysis comparing miRNA-treated cells vs untreated cells. Average±sd of three independent experiments for each cell line are shown. T-test p-value<0.01(**), <0.05(*).

Supplementary Figure 4. RT-PCR analysis of EMT markers. After evaluation of the significant increased expression of miR-502 (A) and miR-532(B) in MDA-MB-231 modulated with miR-502/532 cluster, we analysed the expression of E-Cadherin (Ecad, C), Beta-Catenin (B-cat, D) and Slug (E) expression. Average of three independent experiments is shown (t test, NS= not significant compared to scramble-treated or untreated cells).

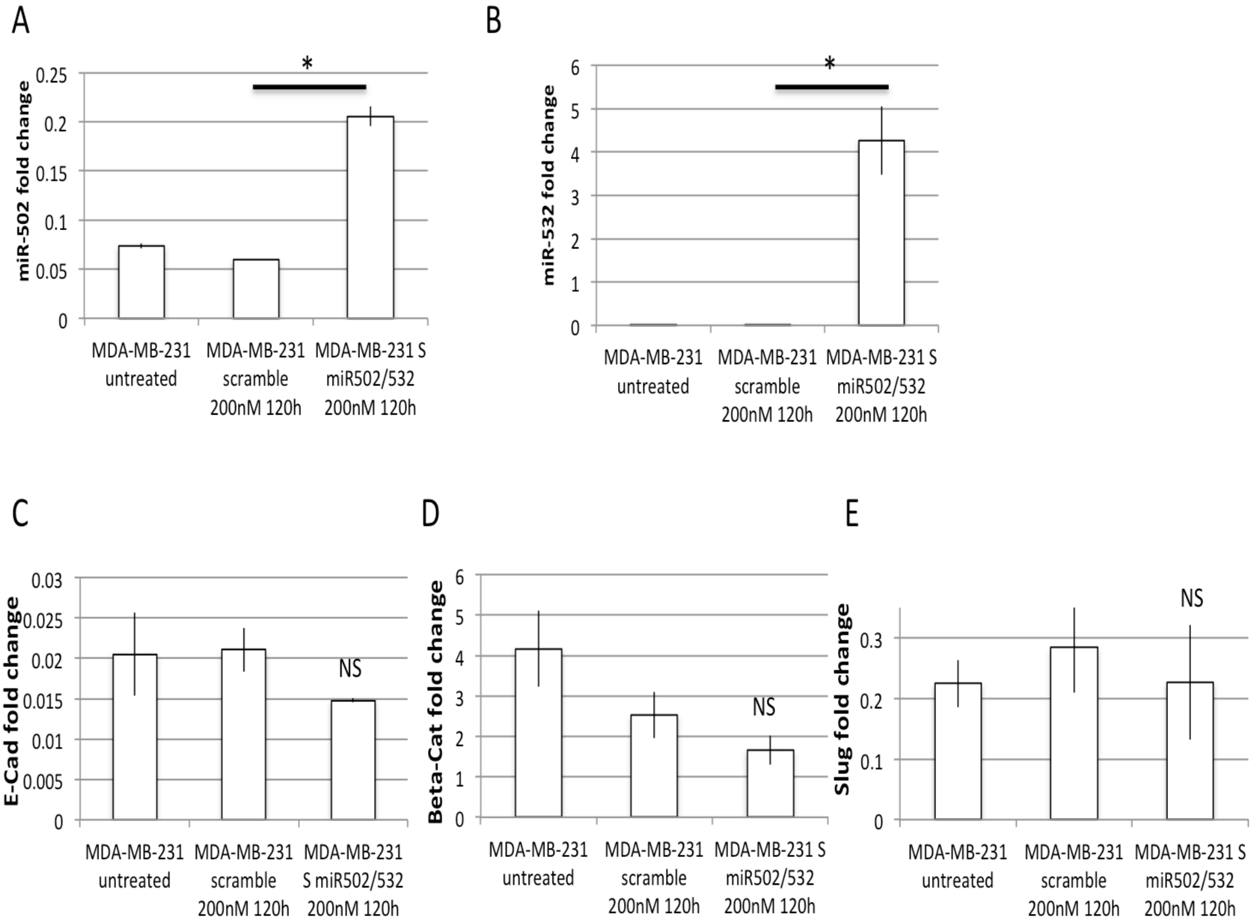

Supplementary Figure 5. Proliferation assay: the cells were treated with 100nM oligonucleotides encoding for sense (S) miR-502-3p (light blue), miR-532-5p (light red) and miR-502/532 cluster (light green) for the indicated times (24, 48, 72, 96h). Count average of three independent experiments is shown (t test compared to scramble-treated cells, *p* value <0.05, *).

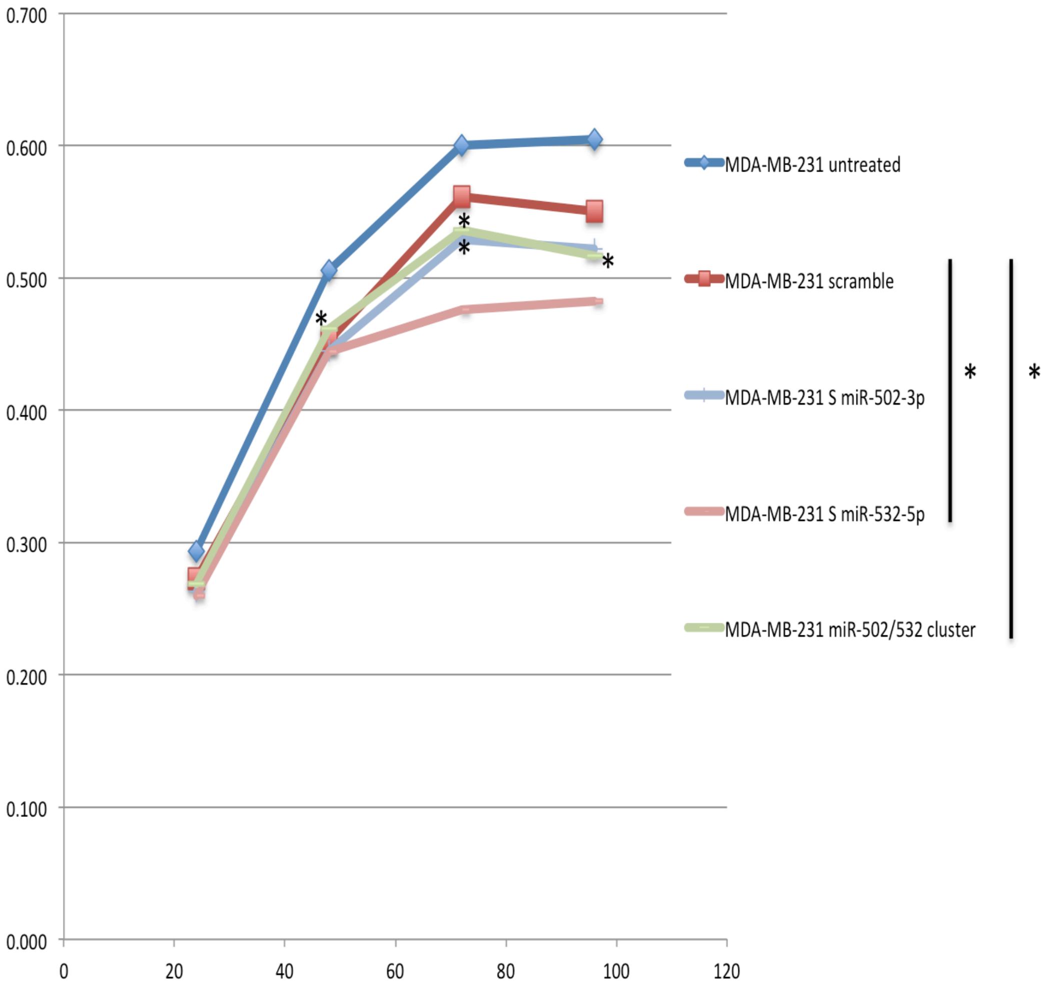

Supplementary Table S1. Sequences of primers designed for RT-PCR analysis of EMT mRNAs and miRNAs.

Oligonucleotide	Sequence (5’->3’)
E-Cadherin (E-Cad)	E-Cad Fw: 5’- GCT GAG CTG GAC AGG GAG GA-3’;
	E-Cad Rew: 5’- ATG GGG GCG TTG TCA TTC AC -3’
N-Cadherin (N-Cad)	N-Cad Fw: 5’- CGA GCC GCC TGC GCT GCC AC -3’;
	N-Cad Rew: 5’- CGC TGC TCT CCG CTC CCC GC -3’
Beta-Catenin (Beta-Cat)	Beta-Cat Fw: 5’- TGG ATG GGC TGC CTC CAG GTG AC 3’;
	Beta-Cat Rew: 5’- ACC AGC CCA CCC CTC GAG CCC -3’
Slug	Slug Fw: 5’- GAC CCT GGT TGC TTC AAG GA -3’;
	Slug Rew: 5’- TGT TGC AGT GAG GGC AAG AA -3’
HPRT	HPRT Fw: 5’- CGA GAT GTG ATG AAG GAG ATG G -3’;
	HPRT Rew: 5’- TGC TTT GAT GTA ATC CAG CAG G -3’
miR-103-3p	Fw: 5’-AGC AGC ATT GTA CAG GGC TAT GA-3’;
miR-214	Fw: 5’-ACA GCA GGC ACA GAC AGG CAG T-3’;
miR-199a-5p	Fw: 5’-CCC AGT GTT CAG ACT ACC TGT TC-3’;
miR-199a-3p	Fw: 5’-ACA GTA GTC TGC ACA TTG GTT AGA CTG G-3’;
miR-532-5p	Fw: 5’-CAT GCC TTG AGT GTA GGA CCG T-3’
miR-502-3p	Fw: 5’-AAT GCA CCT GGG CAA GGA TTC A-3’

Supplementary Table S2. List of genomically co-clustred miRNA output of clustMMRA at step 1 in Breast. The first column reports the miRNA cluster IDs (the list of miRNAs contained in each clusters is reported in the conversion file available at https://github.com/lcan88/Supplementary_miRNA_cluster/blob/master/conversion_file.xlsx), the second the subtype where they are differentially expressed, the third the sign and the fourth the dataset.

MiRNA cluster ID	Subtype	Sign	Breast dataset
cl1017_chr7	Basal	up	TCGA
cl1018_chr7	Basal	up	TCGA
cl1034_chr7	Basal	down	TCGA
cl1039_chr7	Basal	up	Curie
cl1039_chr7	Basal	up	TCGA
cl1071_chr8	Basal	down	TCGA
cl1101_chr8	Basal	up	TCGA
cl1118_chrXXX8	Basal	up	TCGA
cl1145_chr9	Basal	down	Curie
cl1145_chr9	Basal	down	TCGA
cl1147_chr9	Basal	down	Curie
cl1147_chr9	Basal	up	TCGA
cl1157_chr9	Basal	up	TCGA
cl1187_chrX	Basal	up	TCGA
cl1189_chrX	Basal	up	Curie
cl1189_chrX	Basal	up	TCGA
cl1195_chrX	Basal	up	TCGA
cl1196_chrX	Basal	up	TCGA
cl1216_chrX	Basal	up	Curie
cl1216_chrX	Basal	up	TCGA
cl1217_chrX	Basal	down	Curie
cl1217_chrX	Basal	up	TCGA
cl1227_chrX	Basal	up	TCGA
cl1231_chrX	Basal	up	Curie
cl1231_chrX	Basal	up	TCGA
cl1232_chrX	Basal	up	TCGA
cl2_chr1	Basal	up	TCGA
cl233_chr11	Basal	down	TCGA
cl298_chr13	Basal	up	TCGA
cl310_chr13	Basal	up	Curie
cl310_chr13	Basal	up	TCGA
cl34_chr1	Basal	up	TCGA
cl343_chr14	Basal	down	Curie

cl347_chr14	Basal	down	Curie
cl347_chr14	Basal	down	TCGA
cl349_chr14	Basal	down	Curie
cl349_chr14	Basal	down	TCGA
cl416_chr16	Basal	up	TCGA
cl420_chr16	Basal	down	Curie
cl44_chr1	Basal	down	TCGA
cl467_chr17	Basal	down	Curie
cl501_chr17	Basal	up	TCGA
cl563_chr19	Basal	up	TCGA
cl564_chr19	Basal	up	TCGA
cl588_chr19	Basal	down	Curie
cl590_chr19	Basal	up	Curie
cl590_chr19	Basal	up	TCGA
cl722_chr21	Basal	up	TCGA
cl725_chr21	Basal	down	Curie
cl739_chr22	Basal	up	TCGA
cl742_chr22	Basal	up	Curie
cl742_chr22	Basal	up	TCGA
cl746_chr22	Basal	up	TCGA
cl752_chr22	Basal	up	TCGA
cl761_chr22	Basal	down	Curie
cl761_chr22	Basal	down	TCGA
cl786_chr3	Basal	up	Curie
cl81_chr1	Basal	down	Curie
cl81_chr1	Basal	down	TCGA
cl822_chr3	Basal	up	Curie
cl822_chr3	Basal	up	TCGA
cl88_chr1	Basal	up	TCGA
cl904_chr5	Basal	down	TCGA
cl92_chr1	Basal	down	Curie
cl932_chr5	Basal	down	Curie
cl1017_chr7	Her2	up	TCGA
cl1018_chr7	Her2	up	TCGA
cl1145_chr9	Her2	down	TCGA
cl1147_chr9	Her2	up	TCGA
cl1189_chrX	Her2	up	TCGA
cl1217_chrX	Her2	up	TCGA
cl1231_chrX	Her2	up	TCGA
cl2_chr1	Her2	down	TCGA
cl233_chr11	Her2	down	TCGA
cl310_chr13	Her2	up	TCGA
cl34_chr1	Her2	down	Curie
cl349_chr14	Her2	up	TCGA
cl44_chr1	Her2	down	TCGA
cl590_chr19	Her2	down	TCGA

cl742_chr22	Her2	up	TCGA
cl761_chr22	Her2	down	TCGA
cl1017_chr7	Luminal A	down	TCGA
cl1018_chr7	Luminal A	down	TCGA
cl1034_chr7	Luminal A	up	TCGA
cl1039_chr7	Luminal A	down	Curie
cl1039_chr7	Luminal A	down	TCGA
cl1101_chr8	Luminal A	down	TCGA
cl1145_chr9	Luminal A	up	Curie
cl1145_chr9	Luminal A	up	TCGA
cl1147_chr9	Luminal A	down	TCGA
cl1157_chr9	Luminal A	down	TCGA
cl1162_chr9	Luminal A	down	TCGA
cl1187_chrX	Luminal A	down	TCGA
cl1189_chrX	Luminal A	down	Curie
cl1189_chrX	Luminal A	down	TCGA
cl1195_chrX	Luminal A	down	TCGA
cl1196_chrX	Luminal A	down	TCGA
cl1216_chrX	Luminal A	down	Curie
cl1216_chrX	Luminal A	down	TCGA
cl1217_chrX	Luminal A	down	TCGA
cl1227_chrX	Luminal A	down	TCGA
cl1231_chrX	Luminal A	down	TCGA
cl1232_chrX	Luminal A	down	TCGA
cl2_chr1	Luminal A	down	TCGA
cl228_chr11	Luminal A	up	Curie
cl233_chr11	Luminal A	up	Curie
cl233_chr11	Luminal A	up	TCGA
cl238_chr12	Luminal A	down	TCGA
cl298_chr13	Luminal A	down	TCGA
cl310_chr13	Luminal A	down	Curie
cl310_chr13	Luminal A	down	TCGA
cl34_chr1	Luminal A	up	Curie
cl34_chr1	Luminal A	down	TCGA
cl343_chr14	Luminal A	up	Curie
cl347_chr14	Luminal A	up	Curie
cl347_chr14	Luminal A	up	TCGA
cl349_chr14	Luminal A	up	Curie
cl349_chr14	Luminal A	up	TCGA
cl402_chr15	Luminal A	down	TCGA
cl416_chr16	Luminal A	down	TCGA
cl44_chr1	Luminal A	up	Curie
cl44_chr1	Luminal A	up	TCGA
cl451_chr17	Luminal A	down	TCGA
cl454_chr17	Luminal A	up	Curie
cl501_chr17	Luminal A	down	TCGA

cl563_chr19	Luminal A	down	TCGA
cl590_chr19	Luminal A	down	Curie
cl590_chr19	Luminal A	down	TCGA
cl722_chr21	Luminal A	down	TCGA
cl725_chr21	Luminal A	up	Curie
cl739_chr22	Luminal A	down	TCGA
cl742_chr22	Luminal A	down	Curie
cl742_chr22	Luminal A	down	TCGA
cl746_chr22	Luminal A	down	TCGA
cl752_chr22	Luminal A	down	TCGA
cl761_chr22	Luminal A	up	Curie
cl761_chr22	Luminal A	up	TCGA
cl786_chr3	Luminal A	down	Curie
cl81_chr1	Luminal A	up	Curie
cl822_chr3	Luminal A	down	Curie
cl822_chr3	Luminal A	down	TCGA
cl88_chr1	Luminal A	down	TCGA
cl904_chr5	Luminal A	up	TCGA
cl932_chr5	Luminal A	up	Curie
cl96_chr1	Luminal A	down	TCGA
cl1039_chr7	Luminal B	up	TCGA
cl2_chr1	Luminal B	up	TCGA
cl343_chr14	Luminal B	up	Curie
cl904_chr5	Luminal B	down	TCGA
cl92_chr1	Luminal B	up	Curie

Supplementary Table S3. List of genomically co-clustred miRNAs output of clustMMRA at step2 in Breast. The first column reports the miRNA cluster IDs (the list of miRNAs contained in each clusters is reported in the conversion file available at https://github.com/lcan88/Supplementary_miRNA_cluster/blob/master/conversion_file.xlsx), the second the subtype associated to them, then the sign, the sign of the associated gene signature, the minimal number of miRNA-target databases supporting the interaction and finally the dataset.

MiRNA cluster ID	Subtype	Sign miRNA cluster	Sign gene signature	Minimal number of DBs	Breast Dataset
cl81_chr1	Basal	down	up	2	Curie
cl81_chr1	Basal	down	down	2	Curie
cl81_chr1	Basal	down	down	3	Curie
cl81_chr1	Luminal A	up	up	2	Curie
cl81_chr1	Luminal A	up	up	3	Curie
cl228_chr11	Luminal A	up	up	2	Curie
cl347_chr14	Basal	down	up	2	Curie
cl347_chr14	Basal	down	down	2	Curie
cl347_chr14	Luminal A	up	up	2	Curie
cl349_chr14	Basal	down	up	2	Curie
cl349_chr14	Basal	down	down	2	Curie
cl349_chr14	Basal	down	down	3	Curie
cl349_chr14	Luminal A	up	up	2	Curie
cl349_chr14	Luminal A	up	up	3	Curie
cl588_chr19	Basal	down	down	2	Curie
cl590_chr19	Basal	up	up	2	Curie
cl590_chr19	Basal	up	up	4	Curie
cl590_chr19	Basal	up	down	2	Curie
cl590_chr19	Basal	up	down	3	Curie
cl590_chr19	Basal	up	down	4	Curie
cl590_chr19	Luminal A	down	up	2	Curie
cl590_chr19	Luminal A	down	up	3	Curie
cl725_chr21	Basal	down	up	2	Curie
cl725_chr21	Basal	down	up	3	Curie
cl725_chr21	Basal	down	down	2	Curie
cl725_chr21	Luminal A	up	up	2	Curie
cl742_chr22	Basal	up	down	2	Curie
cl742_chr22	Basal	up	down	3	Curie
cl742_chr22	Luminal A	down	up	3	Curie
cl742_chr22	Luminal A	down	up	4	Curie

cl1189_chrX	Basal	up	down	2	Curie
cl1189_chrX	Basal	up	down	3	Curie
cl1189_chrX	Luminal A	down	up	2	Curie
cl1189_chrX	Luminal A	down	up	3	Curie
cl1217_chrX	Basal	down	up	2	Curie
cl1217_chrX	Basal	down	down	2	Curie
cl1217_chrX	Basal	down	down	3	Curie
cl1217_chrX	Basal	down	down	s	Curie
cl2_chr1	Basal	up	up	2	TCGA
cl2_chr1	Basal	up	down	2	TCGA
cl2_chr1	Basal	up	down	3	TCGA
cl2_chr1	Her2	down	down	2	TCGA
cl2_chr1	Luminal A	down	up	2	TCGA
cl2_chr1	Luminal B	up	down	2	TCGA
cl81_chr1	Basal	down	up	2	TCGA
cl81_chr1	Basal	down	down	2	TCGA
cl81_chr1	Basal	down	down	3	TCGA
cl347_chr14	Basal	down	up	2	TCGA
cl347_chr14	Basal	down	down	2	TCGA
cl347_chr14	Luminal A	up	up	2	TCGA
cl349_chr14	Basal	down	up	2	TCGA
cl349_chr14	Basal	down	down	2	TCGA
cl349_chr14	Basal	down	down	3	TCGA
cl349_chr14	Her2	up	up	2	TCGA
cl349_chr14	Her2	up	down	3	TCGA
cl349_chr14	Luminal A	up	up	2	TCGA
cl349_chr14	Luminal A	up	up	3	TCGA
cl416_chr16	Basal	up	down	2	TCGA
cl501_chr17	Basal	up	down	2	TCGA
cl564_chr19	Basal	up	up	2	TCGA
cl564_chr19	Basal	up	down	2	TCGA
cl590_chr19	Basal	up	up	2	TCGA
cl590_chr19	Basal	up	up	4	TCGA
cl590_chr19	Basal	up	down	2	TCGA
cl590_chr19	Basal	up	down	3	TCGA
cl590_chr19	Basal	up	down	4	TCGA
cl590_chr19	Her2	down	up	2	TCGA
cl590_chr19	Luminal A	down	up	2	TCGA
cl590_chr19	Luminal A	down	up	3	TCGA
cl742_chr22	Basal	up	down	2	TCGA
cl742_chr22	Basal	up	down	3	TCGA
cl742_chr22	Luminal A	down	up	3	TCGA
cl742_chr22	Luminal A	down	up	4	TCGA
cl904_chr5	Basal	down	down	2	TCGA
cl904_chr5	Basal	down	down	s	TCGA
cl904_chr5	Luminal A	up	up	2	TCGA

cl904_chr5	Luminal B	down	up	3	TCGA
cl904_chr5	Luminal B	down	up	4	TCGA
cl904_chr5	Luminal B	down	up	s	TCGA
cl904_chr5	Luminal B	down	down	2	TCGA
cl1034_chr7	Basal	down	down	2	TCGA
cl1101_chr8	Basal	up	down	2	TCGA
cl1162_chr9	Luminal A	down	up	3	TCGA
cl1189_chrX	Basal	up	down	2	TCGA
cl1189_chrX	Basal	up	down	3	TCGA
cl1189_chrX	Luminal A	down	up	2	TCGA
cl1189_chrX	Luminal A	down	up	3	TCGA
cl1195_chrX	Basal	up	up	2	TCGA
cl1217_chrX	Basal	up	up	2	TCGA
cl1217_chrX	Basal	up	down	2	TCGA
cl1217_chrX	Basal	up	down	3	TCGA
cl1217_chrX	Luminal A	down	up	3	TCGA
cl1217_chrX	Luminal A	down	down	4	TCGA
cl1217_chrX	Luminal A	down	down	s	TCGA

Supplementary Table S4. List of genomically co-clustred miRNA output of clustMMRA at step 1 in CRC. The first column reports the miRNA cluster IDs (the list of miRNAs contained in each clusters is reported in the conversion file available at https://github.com/lcan88/Supplementary_miRNA_cluster/blob/master/conversion_file.xlsx), the second the subtype where they are differentially expressed, the third the sign

MiRNA cluster ID	CRCA Subtype	Sign	Dataset
cl96_chr1	1	down	CRC TCGA
cl347_chr14	1	up	CRC TCGA
cl590_chr19	1	up	CRC TCGA
cl591_chr19	1	down	CRC TCGA
cl1034_chr7	1	down	CRC TCGA
cl1217_chrX	1	up	CRC TCGA
cl1224_chrX	1	up	CRC TCGA
cl1226_chrX	1	up	CRC TCGA
cl96_chr1	2	down	CRC TCGA
cl349_chr14	2	up	CRC TCGA
cl564_chr19	2	down	CRC TCGA
cl591_chr19	2	down	CRC TCGA
cl611_chr2	2	down	CRC TCGA
cl1092_chr8	2	down	CRC TCGA
cl1228_chrX	2	down	CRC TCGA
cl51_chr1	3	down	CRC TCGA
cl347_chr14	3	down	CRC TCGA
cl564_chr19	3	down	CRC TCGA
cl590_chr19	3	down	CRC TCGA
cl1034_chr7	3	up	CRC TCGA
cl1118_chrXXX8	3	up	CRC TCGA
cl1195_chrX	3	down	CRC TCGA
cl1224_chrX	3	up	CRC TCGA
cl51_chr1	4	up	CRC TCGA
cl591_chr19	4	up	CRC TCGA
cl2_chr1	5	up	CRC TCGA
cl51_chr1	5	down	CRC TCGA
cl96_chr1	5	up	CRC TCGA
cl564_chr19	5	up	CRC TCGA
cl590_chr19	5	down	CRC TCGA

cl611_chr2	5	up	CRC TCGA
cl742_chr22	5	up	CRC TCGA
cl1034_chr7	5	up	CRC TCGA
cl1092_chr8	5	up	CRC TCGA
cl1118_chrXXX8	5	down	CRC TCGA
cl1190_chrX	5	down	CRC TCGA
cl1217_chrX	5	down	CRC TCGA
cl1224_chrX	5	down	CRC TCGA
cl1226_chrX	5	down	CRC TCGA

Supplementary Table S5. List of genomically co-clustred miRNAs output of clustMMRA at step2 in CRC. The first column reports the miRNA cluster IDs (the list of miRNAs contained in each clusters is reported in the conversion file available at https://github.com/lcan88/Supplementary_miRNA_cluster/blob/master/conversion_file.xlsx), the second the subtype associated to them, then the sign, the sign of the associated gene signature, the minimal number of miRNA-target databases supporting the interaction.

MiRNA cluster ID	CRCA Subtype	Sign miRNA cluster	Sign gene signature	Minimal number of DBs
cl96_chr1	1	down	up	3
cl347_chr14	1	up	up	2
cl347_chr14	1	up	down	2
cl590_chr19	1	up	down	2
cl590_chr19	1	up	down	3
cl590_chr19	1	up	down	4
cl591_chr19	1	down	down	2
cl591_chr19	1	down	down	3
cl591_chr19	1	down	down	4
cl1034_chr7	1	down	up	2
cl1034_chr7	1	down	down	2
cl1034_chr7	1	down	down	4
cl1217_chrX	1	up	down	2
cl1217_chrX	1	up	down	3
cl1224_chrX	1	up	down	2
cl1224_chrX	1	up	down	3
cl1226_chrX	1	up	down	2
cl96_chr1	2	down	down	2
cl96_chr1	2	down	down	3
cl349_chr14	2	up	up	2
cl349_chr14	2	up	up	3
cl349_chr14	2	up	down	2
cl349_chr14	2	up	down	3
cl349_chr14	2	up	down	4
cl564_chr19	2	down	down	2
cl564_chr19	2	down	down	3
cl564_chr19	2	down	down	4
cl591_chr19	2	down	down	2
cl591_chr19	2	down	down	3
cl611_chr2	2	down	up	2

cl611_chr2	2	down	down	2
cl611_chr2	2	down	down	3
cl1092_chr8	2	down	up	2
cl1092_chr8	2	down	down	2
cl1092_chr8	2	down	down	3
cl1228_chrX	2	down	down	2
cl51_chr1	3	down	up	2
cl51_chr1	3	down	down	2
cl51_chr1	3	down	down	3
cl51_chr1	3	down	down	4
cl347_chr14	3	down	down	2
cl564_chr19	3	down	down	2
cl590_chr19	3	down	down	2
cl590_chr19	3	down	down	3
cl590_chr19	3	down	down	4
cl1034_chr7	3	up	up	2
cl1195_chrX	3	down	down	2
cl1224_chrX	3	up	down	2
cl51_chr1	4	up	up	2
cl51_chr1	4	up	up	3
cl51_chr1	4	up	up	4
cl51_chr1	4	up	down	2
cl51_chr1	4	up	down	4
cl591_chr19	4	up	up	2
cl591_chr19	4	up	up	3
cl591_chr19	4	up	up	4
cl591_chr19	4	up	down	2
cl591_chr19	4	up	down	3
cl2_chr1	5	up	up	2
cl2_chr1	5	up	up	3
cl51_chr1	5	down	up	2
cl51_chr1	5	down	up	3
cl51_chr1	5	down	up	4
cl51_chr1	5	down	up	s
cl51_chr1	5	down	down	2
cl96_chr1	5	up	up	2
cl96_chr1	5	up	up	3
cl564_chr19	5	up	up	2
cl564_chr19	5	up	up	3
cl564_chr19	5	up	up	4
cl564_chr19	5	up	down	2
cl590_chr19	5	down	up	2
cl590_chr19	5	down	up	3
cl590_chr19	5	down	up	4
cl611_chr2	5	up	up	2
cl611_chr2	5	up	up	3

cl611_chr2	5	up	down	2
cl742_chr22	5	up	up	2
cl742_chr22	5	up	up	3
cl742_chr22	5	up	up	4
cl1034_chr7	5	up	up	2
cl1034_chr7	5	up	up	3
cl1034_chr7	5	up	down	2
cl1092_chr8	5	up	up	2
cl1092_chr8	5	up	up	3
cl1092_chr8	5	up	down	2
cl1190_chrX	5	down	up	2
cl1190_chrX	5	down	up	3
cl1190_chrX	5	down	up	4
cl1190_chrX	5	down	down	2
cl1217_chrX	5	down	up	2
cl1217_chrX	5	down	up	3
cl1224_chrX	5	down	up	2
cl1224_chrX	5	down	down	2
cl1224_chrX	5	down	down	3
cl1226_chrX	5	down	up	2
cl1226_chrX	5	down	up	3

Supplementary Table S6. List of genomically co-clustred miRNAs output of clustMMRA in CRC. The first column reports the miRNA cluster IDs (the list of miRNAs contained in each clusters is reported in the conversion file available at https://github.com/lcan88/Supplementary_miRNA_cluster/blob/master/conversion_file.xlsx), the second the subtype associated to them, then the sign, the sign of the associated gene signature..

MiRNA cluster ID	CRCA Subtype	Sign miRNA cluster	Sign gene signature
cl1034_chr7	5	up	up
cl1034_chr7	5	up	up
cl1190_chrX	5	down	up
cl1190_chrX	5	down	up
cl1190_chrX	5	down	up
cl1190_chrX	5	down	down
cl1195_chrX	3	down	down
cl1217_chrX	1	up	down
cl1217_chrX	1	up	down
cl1217_chrX	5	down	up
cl1217_chrX	5	down	up
cl1226_chrX	1	up	down
cl1226_chrX	5	down	up
cl1226_chrX	5	down	up
cl2_chr1	5	up	up
cl2_chr1	5	up	up
cl347_chr14	1	up	up
cl349_chr14	2	up	up
cl349_chr14	2	up	up
cl349_chr14	2	up	down
cl349_chr14	2	up	down
cl349_chr14	2	up	down
cl96_chr1	1	down	up
cl96_chr1	2	down	down
cl96_chr1	2	down	down
cl96_chr1	5	up	up
cl96_chr1	5	up	up

Supplementary Table S7. Pathways enriched in the regulons of the 4 miRNA clusters output of clustMMRA in both Curie and TCGA datasets.

MiRNA cluster ID	MsigDB gene set name	# Genes in Gene Set (K)	# Genes in Overlap (k)	k/K	p-value	FDR q-value
miR-532/502	SOTIRIOU_BREAST_CANCER_GRADE_1_VS_3_UP	151	34	0.2252	1.27E-66	2.26E-62
miR-532/502	GOBERT_OLIGODENDROCYTE_DIFFERENTIATION_UP	570	43	0.0754	7.11E-64	6.32E-60
miR-532/502	MODULE_54	263	32	0.1217	3.25E-53	1.92E-49
miR-532/502	ROSTY_CERVICAL_CANCER_PROLIFERATION_CLUSTER	140	28	0.2	8.17E-53	3.63E-49
miR-532/502	CHANG_CYCLING_GENES	148	28	0.1892	4.54E-52	1.62E-48
miR-532/502	KOBAYASHI_EGFR_SIGNALING_24HR_DN	251	31	0.1235	1.11E-51	3.28E-48
miR-532/502	DUTERTRE_ESTRADIOL_RESPONSE_24HR_UP	324	32	0.0988	3.59E-50	9.11E-47
miR-532/502	CHIANG_LIVER_CANCER_SUBCLASS_PROLIFERATION_UP	178	28	0.1573	1.26E-49	2.81E-46
miR-532/502	HORIUCHI_WTAP_TARGETS_DN	310	31	0.1	1.07E-48	2.12E-45
miR-532/502	BENPORATH_CYCLING_GENES	648	36	0.0556	1.24E-47	2.21E-44
miR-532/502	KINSEY_TARGETS_OF_EWSR1_FLII_FUSION_UP	1278	42	0.0329	7.38E-47	1.19E-43
miR-532/502	GSE15750_DAY6_VS_DAY10_EFF_CD8_TCELL_UP	200	27	0.135	7.05E-46	1.04E-42
miR-532/502	MARSON_BOUND_BY_E2F4_UNSTIMULATED	728	36	0.0495	8.64E-46	1.18E-42
miR-532/502	NUYTTEN_EZH2_TARGETS_DN	1024	39	0.0381	1.46E-45	1.86E-42
miR-532/502	BASAKI_YBX1_TARGETS_UP	290	29	0.1	2.05E-45	2.43E-42
miR-532/502	GO_MITOTIC_CELL_CYCLE	766	36	0.047	5.48E-45	6.09E-42
miR-532/502	GSE15750_DAY6_VS_DAY10_TRAF6KO_EFF_CD8_TCELL_UP	200	26	0.13	1.11E-43	1.16E-40
miR-532/502	SHEDDEN_LUNG_CANCER_POOR_SURVIVAL_A6	456	31	0.068	2.47E-43	2.44E-40
miR-532/502	GO_CELL_CYCLE	1316	39	0.0296	2.50E-41	2.34E-38
miR-532/502	GNF2_CDC20	56	19	0.3393	1.12E-40	9.99E-38
miR-532/502	GNF2_CCNB2	57	19	0.3333	1.68E-40	1.43E-37
miR-532/502	GO_CELL_CYCLE_PROCESS	1081	36	0.0333	1.34E-39	1.09E-36
miR-532/502	LEE_EARLY_T_LYMPHOCYTE_UP	107	21	0.1963	3.14E-39	2.43E-36
miR-532/502	DODD_NASOPHARYNGEAL_CARCINOMA_DN	1375	38	0.0276	5.36E-39	3.97E-36
miR-532/502	GNF2_CCNA2	68	19	0.2794	8.76E-39	6.23E-36
miR-532/502	VECCHI_GASTRIC_CANCER_EARLY_UP	430	28	0.0651	2.05E-38	1.40E-35
miR-532/502	GRAHAM_CML_DIVIDING_VS_NORMAL_QUIESCENT_UP	181	23	0.1271	2.60E-38	1.71E-35
miR-532/502	GSE24634_TEFF_VS_TCONV_DAY7_IN_CULTURE_UP	200	23	0.115	2.93E-37	1.80E-34
miR-532/502	HALLMARK_E2F_TARGETS	200	23	0.115	2.93E-37	1.80E-34
miR-532/502	GNF2_CDC2	62	18	0.2903	4.21E-37	2.49E-34
miR-532/502	GO_MITOTIC_NUCLEAR_DIVISION	361	26	0.072	9.67E-37	5.54E-34
miR-532/502	ODONNELL_TFRC_TARGETS_DN	139	21	0.1511	1.23E-36	6.84E-34
miR-532/502	KANG_DOXORUBICIN_RESISTANCE_UP	54	17	0.3148	9.19E-36	4.95E-33
miR-532/502	GSE21063_WT_VS_NFATC1_KO_8H_ANTI_IGM_STIM_BCELL_UP	200	22	0.11	3.54E-35	1.80E-32
miR-532/502	GSE36476_CTRL_VS_TSST_ACT_72H_MEMORY_CD4_TCELL_YOUNG_DN	200	22	0.11	3.54E-35	1.80E-32
miR-532/502	GSE24634_TREG_VS_TCONV_POST_DAY7_IL4_CONVERSION_UP	200	21	0.105	3.97E-33	1.96E-30
miR-532/502	GO_ORGANELLE_FISSION	496	26	0.0524	4.24E-33	2.04E-30
miR-532/502	GO_CELL_DIVISION	460	25	0.0543	3.47E-32	1.62E-29
miR-532/502	GNF2_CENPF	62	16	0.2581	4.40E-32	2.01E-29
miR-532/502	GRAHAM_NORMAL_QUIESCENT_VS_NORMAL_DIVIDING_DN	87	17	0.1954	9.30E-32	4.13E-29
miR-532/502	GNF2_HMMR	47	15	0.3191	1.01E-31	4.37E-29
miR-532/502	GSE37532_WT_VS_PPARG_KO_VISCERAL_ADIPOSE_TISSUE_TREG_UP	200	20	0.1	4.16E-31	1.72E-28
miR-532/502	GSE39556_CD8A_DC_VS_NK_CELL_MOUSE_3H_POST_POLYIC_INJ_UP	200	20	0.1	4.16E-31	1.72E-28
miR-532/502	CROONQUIST_NRAS_SIGNALING_DN	72	16	0.2222	6.56E-31	2.65E-28
miR-532/502	KONG_E2F3_TARGETS	97	17	0.1753	7.02E-31	2.77E-28
miR-532/502	GNF2_RRM2	40	14	0.35	2.53E-30	9.76E-28
miR-532/502	FARMER_BREAST_CANCER_BASAL_VS_LULMINAL	330	22	0.0667	3.05E-30	1.16E-27
miR-532/502	GO_CHROMOSOME_SEGREGATION	272	21	0.0772	3.16E-30	1.17E-27
miR-532/502	PUJANA_BRCA2_PCC_NETWORK	423	23	0.0544	1.44E-29	5.23E-27
miR-532/502	GSE45365_WT_VS_IFNAR_KO_BCELL_MCMV_INFECTION_DN	196	19	0.0969	2.71E-29	9.65E-27
miR-532/502	GSE39110_DAY3_VS_DAY6_POST_IMMUNIZATION_CD8_TCELL_DN	200	19	0.095	4.04E-29	1.38E-26
miR-532/502	HALLMARK_G2M_CHECKPOINT	200	19	0.095	4.04E-29	1.38E-26
miR-532/502	LINDGREN_BLADDER_CANCER_CLUSTER_1_DN	378	22	0.0582	6.33E-29	2.12E-26
miR-532/502	CROONQUIST_IL6_DEPRIVATION_DN	98	16	0.1633	1.47E-28	4.83E-26
miR-532/502	WONG_EMBRYONIC_STEM_CELL_CORE	335	21	0.0627	2.73E-28	8.84E-26
miR-532/502	GSE14415_INDUCED_VS_NATURAL_TREG_DN	178	18	0.1011	4.14E-28	1.31E-25
miR-532/502	BLUM_RESPONSE_TO_SALIRASIB_DN	342	21	0.0614	4.25E-28	1.32E-25
miR-532/502	GSE14415_NATURAL_TREG_VS_TCONV_DN	180	18	0.1	5.10E-28	1.56E-25
miR-532/502	PUJANA_CHEK2_PCC_NETWORK	779	26	0.0334	5.18E-28	1.56E-25
miR-532/502	GSE13547_CTRL_VS_ANTI_IGM_STIM_BCELL_12H_UP	182	18	0.0989	6.27E-28	1.86E-25
miR-532/502	GSE45365_HEALTHY_VS_MCMV_INFECTION_CD11B_DC_DN	197	18	0.0914	2.75E-27	8.01E-25
miR-532/502	MITSIADES_RESPONSE_TO_APLIDIN_DN	249	19	0.0763	2.95E-27	8.45E-25
miR-532/502	GSE30962_PRIMARY_VS_SECONDARY_ACUTE_LCMV_INF_CD8_TCELL_UP	199	18	0.0905	3.32E-27	9.36E-25
miR-532/502	NAKAYAMA_SOFT_TISSUE_TUMORS_PCA2_UP	87	15	0.1724	3.37E-27	9.37E-25
miR-532/502	GSE2405_S_AUREUS_VS_UNTREATED_NEUTROPHIL_DN	200	18	0.09	3.64E-27	9.81E-25
miR-532/502	GSE36476_CTRL_VS_TSST_ACT_72H_MEMORY_CD4_TCELL_OLD_DN	200	18	0.09	3.64E-27	9.81E-25
miR-532/502	WINNEPENNINCKX_MELANOMA_METASTASIS_UP	162	17	0.1049	7.43E-27	1.97E-24
miR-532/502	LINDGREN_BLADDER_CANCER_CLUSTER_3_UP	329	20	0.0608	1.13E-26	2.96E-24
miR-532/502	CASORELLI_ACUTE_PROMYELOCYTIC_LEUKEMIA_DN	663	24	0.0362	1.23E-26	3.17E-24
miR-532/502	GNF2_BUB1B	49	13	0.2653	2.24E-26	5.68E-24
miR-532/502	BURTON_ADIPOGENESIS_3	101	15	0.1485	3.75E-26	9.40E-24
miR-532/502	GO_NUCLEAR_CHROMOSOME_SEGREGATION	228	18	0.0789	4.12E-26	1.02E-23
miR-532/502	POOLA_INVASIVE_BREAST_CANCER_UP	288	19	0.066	4.94E-26	1.20E-23
miR-532/502	CAIRO_HEPATOBLASTOMA_CLASSES_UP	605	23	0.038	5.41E-26	1.30E-23
miR-532/502	GNF2_ESPL1	35	12	0.3429	5.62E-26	1.33E-23

miR-532/502	RUIZ_TNC_TARGETS_DN	142	16	0.1127	7.99E-26	1.87E-23
miR-532/502	CHICAS_RB1_TARGETS_GROWING	243	18	0.0741	1.33E-25	3.08E-23
miR-532/502	BERENJENO_TRANSFORMED_BY_RHOA_UP	536	22	0.041	1.41E-25	3.21E-23
miR-532/502	BENPORATH_PROLIFERATION	147	16	0.1088	1.43E-25	3.21E-23
miR-532/502	GSE36476_CTRL_VS_TSST_ACT_40H_MEMORY_CD4_TCELL_YOUNG_DN	200	17	0.085	3.03E-25	6.74E-23
miR-532/502	GAVIN_FOXP3_TARGETS_CLUSTER_P6	91	14	0.1538	1.10E-24	2.41E-22
miR-532/502	FOURNIER_ACINAR_DEVELOPMENT_LATE_2	277	18	0.065	1.47E-24	3.15E-22
miR-532/502	ZHANG_TLX_TARGETS_60HR_DN	277	18	0.065	1.47E-24	3.15E-22
miR-532/502	ZHOU_CELL_CYCLE_GENES_IN_IR_RESPONSE_24HR	128	15	0.1172	1.62E-24	3.42E-22
miR-532/502	GNF2_PCNA	68	13	0.1912	2.61E-24	5.47E-22
miR-532/502	GO_SISTER_CHROMATID_SEGREGATION	176	16	0.0909	2.84E-24	5.87E-22
miR-532/502	SARRIO_EPITHELIAL_MESENCHYMAL_TRANSITION_UP	180	16	0.0889	4.12E-24	8.42E-22
miR-532/502	GSE14415_TCONV_VS_FOXP3_KO_INDUCED_TREG_DN	183	16	0.0874	5.41E-24	1.09E-21
miR-532/502	ZHENG_GLIOBLASTOMA_PLASTICITY_UP	250	17	0.068	1.47E-23	2.94E-21
miR-532/502	GSE45365_WT_VS_IFNAR_KO_BCELL_DN	195	16	0.0821	1.54E-23	3.04E-21
miR-532/502	GNF2_MCM4	53	12	0.2264	1.76E-23	3.44E-21
miR-532/502	GSE45365_HEALTHY_VS_MCMV_INFECTION_CD11B_DC_IFNAR_KO_DN	197	16	0.0812	1.82E-23	3.52E-21
miR-532/502	GSE24634_IL4_VS_CTRL_TREATED_NAIVE_CD4_TCELL_DAY7_UP	200	16	0.08	2.33E-23	4.32E-21
miR-532/502	GSE25088_WT_VS_STAT6_KO_MACROPHAGE_IL4_STIM_DN	200	16	0.08	2.33E-23	4.32E-21
miR-532/502	GSE29614_CTRL_VS_DAY7_TIV_FLU_VACCINE_PBMC_DN	200	16	0.08	2.33E-23	4.32E-21
miR-532/502	GSE33292_WT_VS_TCF1_KO_DN3_THYMOCYTE_DN	200	16	0.08	2.33E-23	4.32E-21
miR-532/502	WHITEFORD_PEDIATRIC_CANCER_MARKERS	116	14	0.1207	4.04E-23	7.40E-21
miR-532/502	MOLENAAR_TARGETS_OF_CCND1_AND_CDK4_DN	58	12	0.2069	5.84E-23	1.06E-20
miR-532/502	REACTOME_CELL_CYCLE	421	19	0.0451	7.06E-23	1.27E-20
miR-532/502	WHITFIELD_CELL_CYCLE_G2_M	216	16	0.0741	8.22E-23	1.46E-20
miR-493/136	SCHUETZ_BREAST_CANCER_DUCTAL_INVASIVE_UP	351	71	0.2023	4.35E-115	7.74E-111
miR-493/136	MODULE_47	225	53	0.2356	4.68E-88	4.16E-84
miR-493/136	BOQUEST_STEM_CELL_UP	260	54	0.2077	1.70E-86	1.01E-82
miR-493/136	ANASTASSIOU_MULTICANCER_INVASIVENESS_SIGNATURE	64	39	0.6094	1.55E-84	6.89E-81
miR-493/136	GO_EXTRACELLULAR_MATRIX	426	58	0.1362	9.35E-82	3.33E-78
miR-493/136	HALLMARK_EPITHELIAL_MESENCHYMAL_TRANSITION	200	48	0.24	7.70E-80	2.28E-76
miR-493/136	NABA_CORE_MATRISOME	275	50	0.1818	1.31E-76	3.33E-73
miR-493/136	GO_PROTEINACEOUS_EXTRACELLULAR_MATRIX	356	53	0.1489	1.80E-76	4.00E-73
miR-493/136	NABA_MATRISOME	1028	64	0.0623	1.90E-68	3.75E-65
miR-493/136	GO_EXTRACELLULAR_STRUCTURE_ORGANIZATION	304	42	0.1382	1.49E-58	2.65E-55
miR-493/136	ONDER_CDH1_TARGETS_2_UP	256	37	0.1445	3.43E-52	5.54E-49
miR-493/136	MODULE_1	368	40	0.1087	2.56E-51	3.79E-48
miR-493/136	LINDGREN_BLADDER_CANCER_CLUSTER_2B	392	38	0.0969	9.72E-47	1.33E-43
miR-493/136	LIM_MAMMARY_STEM_CELL_UP	489	40	0.0818	3.03E-46	3.85E-43
miR-493/136	FARMER_BREAST_CANCER_CLUSTER_4	19	18	0.9474	1.54E-45	1.83E-42
miR-493/136	TURASHVILI_BREAST_LOBULAR_CARCINOMA_VS_DUCTAL_NORMAL_UP	69	24	0.3478	4.05E-44	4.50E-41
miR-493/136	CHICAS_RB1_TARGETS_CONFLUENT	567	40	0.0705	1.21E-43	1.27E-40
miR-493/136	NABA_ECM_GLYCOPROTEINS	196	30	0.1531	4.98E-43	4.92E-40
miR-493/136	GO_EXTRACELLULAR_MATRIX_COMPONENT	125	26	0.208	4.50E-41	4.21E-38
miR-493/136	GO_EXTRACELLULAR_SPACE	1376	49	0.0356	1.45E-39	1.29E-36
miR-493/136	TURASHVILI_BREAST_LOBULAR_CARCINOMA_VS_LOBULAR_NORMAL_DN	74	22	0.2973	1.05E-38	8.69E-36
miR-493/136	PICCALUGA_ANGIOIMMUNOBLASTIC_LYMPHOMA_UP	205	28	0.1366	1.08E-38	8.69E-36
miR-493/136	CHARAFE_BREAST_CANCER_LUMINAL_VS_MESENCHYMAL_DN	460	34	0.0739	1.16E-37	8.97E-35
miR-493/136	MODULE_122	141	25	0.1773	1.30E-37	9.63E-35
miR-493/136	BERENJENO_TRANSFORMED_BY_RHOA_DN	394	31	0.0787	3.63E-35	2.58E-32
miR-493/136	SWEET_LUNG_CANCER_KRAS_DN	435	31	0.0713	8.03E-34	5.49E-31
miR-493/136	VECCHI_GASTRIC_CANCER_ADVANCED_VS_EARLY_UP	175	24	0.1371	2.88E-33	1.90E-30
miR-493/136	KIM_GLIS2_TARGETS_UP	84	20	0.2381	4.82E-33	3.06E-30
miR-493/136	LIU_PROSTATE_CANCER_DN	481	31	0.0644	1.82E-32	1.12E-29
miR-493/136	WEST_ADRENOCORTICAL_TUMOR_DN	546	32	0.0586	3.33E-32	1.98E-29
miR-493/136	CLASPER_LYMPHATIC_VESSELS_DURING_METASTASIS_DN	36	16	0.4444	8.51E-32	4.88E-29
miR-493/136	REN_ALVEOLAR_RHABDOMYOSARCOMA_DN	408	29	0.0711	1.31E-31	7.28E-29
miR-493/136	WONG_ENDMETRIUM_CANCER_DN	82	19	0.2317	3.51E-31	1.89E-28
miR-493/136	GO_BIOLOGICAL_ADHESION	1032	38	0.0368	7.72E-31	3.93E-28
miR-493/136	MODULE_2	384	28	0.0729	7.74E-31	3.93E-28
miR-493/136	IZADPANAH_STEM_CELL_ADIPOSE_VS_BONE_DN	108	20	0.1852	1.24E-30	6.15E-28
miR-493/136	GNF2_CDH11	25	13	0.52	3.38E-27	1.62E-24
miR-493/136	GNF2_PTX3	36	14	0.3889	6.39E-27	2.99E-24
miR-493/136	SERVITJA_ISLET_HNF1A_TARGETS_UP	163	20	0.1227	7.95E-27	3.62E-24
miR-493/136	DURAND_STROMA_S_UP	297	23	0.0774	5.07E-26	2.25E-23
miR-493/136	CHEN_METABOLIC_SYNDROM_NETWORK	1210	36	0.0298	5.24E-26	2.27E-23
miR-493/136	RODWELL_AGING_KIDNEY_NO_BLOOD_UP	222	21	0.0946	1.12E-25	4.76E-23
miR-493/136	MODULE_12	360	24	0.0667	1.47E-25	6.09E-23
miR-493/136	SENESE_HDAC1_AND_HDAC2_TARGETS_DN	232	20	0.0862	1.14E-23	4.52E-21
miR-493/136	GO_EXTRACELLULAR_MATRIX_STRUCTURAL_CONSTITUENT	76	15	0.1974	1.14E-23	4.52E-21
miR-493/136	WANG_SMARCE1_TARGETS_UP	280	21	0.075	1.58E-23	6.10E-21
miR-493/136	RB_P107_DN.V1_UP	140	17	0.1214	7.32E-23	2.77E-20
miR-493/136	PID_INTEGRIN1_PATHWAY	66	14	0.2121	1.21E-22	4.48E-20
miR-493/136	CUI_TCF21_TARGETS_2_UP	428	23	0.0537	2.18E-22	7.93E-20
miR-493/136	GO_REGULATION_OF_MULTICELLULAR_ORGANISMAL_DEVELOPMENT	1672	37	0.0221	2.51E-22	8.94E-20
miR-493/136	MODULE_5	434	23	0.053	2.99E-22	1.04E-19
miR-493/136	KINSEY_TARGETS_OF_EWSR1_FLII_FUSION_DN	329	21	0.0638	4.66E-22	1.59E-19
miR-493/136	CHICAS_RB1_TARGETS_GROWING	243	19	0.0782	1.02E-21	3.43E-19
miR-493/136	LEE_BMP2_TARGETS_UP	745	27	0.0362	1.05E-21	3.44E-19
miR-493/136	BERTUCCI_MEDULLARY_VS_DUCTAL_BREAST_CANCER_DN	169	17	0.1006	2.00E-21	6.47E-19
miR-493/136	CHIANG_LIVER_CANCER_SUBCLASS_CTNNB1_DN	170	17	0.1	2.22E-21	7.05E-19
miR-493/136	LINDVALL_IMMORTALIZED_BY_TERT_DN	80	14	0.175	2.28E-21	7.11E-19
miR-493/136	WESTON_VEGFA_TARGETS_6HR	59	13	0.2203	2.42E-21	7.41E-19
miR-493/136	ESC_V6.5_UP_EARLY.V1_DN	172	17	0.0988	2.72E-21	8.20E-19

miR-493/136	SCHAEFFER_PROSTATE_DEVELOPMENT_48HR_DN	428	22	0.0514	4.99E-21	1.48E-18
miR-493/136	IGLESIAS_E2F_TARGETS_UP	151	16	0.106	1.35E-20	3.93E-18
miR-493/136	RIGGI_EWING_SARCOMA_PROGENITOR_DN	191	17	0.089	1.68E-20	4.81E-18
miR-493/136	HOSHIDA_LIVER_CANCER_SUBCLASS_S1	237	18	0.0759	2.15E-20	6.03E-18
miR-493/136	GO_BASEMENT_MEMBRANE	93	14	0.1505	2.17E-20	6.03E-18
miR-493/136	SASAI_RESISTANCE_TO_NEOPLASTIC_TRANSFROMATION	50	12	0.24	2.76E-20	7.48E-18
miR-493/136	WANG_MLL_TARGETS	289	19	0.0657	2.78E-20	7.48E-18
miR-493/136	MODULE_38	465	22	0.0473	2.96E-20	7.85E-18
miR-493/136	LIEN_BREAST_CARCINOMA_METAPLASTIC	35	11	0.3143	3.64E-20	9.52E-18
miR-493/136	GO_GLYCOSAMINOGLYCAN_BINDING	205	17	0.0829	5.68E-20	1.46E-17
miR-493/136	THUM_MIR21_TARGETS_HEART_DISEASE_UP	17	9	0.5294	2.96E-19	7.46E-17
miR-493/136	ONDER_CDH1_SIGNALING_VIA_CTNNB1	83	13	0.1566	2.98E-19	7.46E-17
miR-493/136	BRUINS_UVC_RESPONSE_LATE	1137	29	0.0255	3.93E-19	9.71E-17
miR-493/136	LANDIS_ERBB2_BREAST_TUMORS_324_DN	149	15	0.1007	4.92E-19	1.20E-16
miR-493/136	REACTOME_EXTRACELLULAR_MATRIX_ORGANIZATION	87	13	0.1494	5.70E-19	1.37E-16
miR-493/136	TURASHVILI_BREAST_DUCTAL_CARCINOMA_VS_DUCTAL_NORMAL_UP	44	11	0.25	6.55E-19	1.55E-16
miR-493/136	GO_COLLAGEN_TRIMER	88	13	0.1477	6.68E-19	1.56E-16
miR-493/136	GO_REGULATION_OF_CELL_DIFFERENTIATION	1492	32	0.0214	6.90E-19	1.59E-16
miR-493/136	GO_VASCULATURE_DEVELOPMENT	469	21	0.0448	6.97E-19	1.59E-16
miR-493/136	GO_CIRCULATORY_SYSTEM_DEVELOPMENT	788	25	0.0317	9.11E-19	2.05E-16
miR-493/136	ZHU_CMV_24_HR_DN	91	13	0.1429	1.06E-18	2.35E-16
miR-493/136	WONG_ADULT_TISSUE_STEM_MODULE	721	24	0.0333	1.63E-18	3.58E-16
miR-493/136	LANDIS_BREAST_CANCER_PROGRESSION_DN	70	12	0.1714	2.30E-18	4.94E-16
miR-493/136	MIKKELSEN_MEF_LCP_WITH_H3K4ME3	128	14	0.1094	2.33E-18	4.94E-16
miR-493/136	ZHU_CMV_ALL_DN	128	14	0.1094	2.33E-18	4.94E-16
miR-493/136	GU_PDEF_TARGETS_UP	71	12	0.169	2.77E-18	5.78E-16
miR-493/136	SENESE_HDAC2_TARGETS_DN	133	14	0.1053	4.06E-18	8.39E-16
miR-493/136	NABA_PROTEOGLYCANS	35	10	0.2857	5.87E-18	1.20E-15
miR-493/136	GO_REGULATION_OF_CELLULAR_COMPONENT_MOVEMENT	771	24	0.0311	7.43E-18	1.50E-15
miR-493/136	GO_COMPLEX_OF_COLLAGEN_TRIMERS	23	9	0.3913	9.80E-18	1.96E-15
miR-493/136	CHIARADONNA_NEOPLASTIC_TRANSFORMATION_KRAS_DN	142	14	0.0986	1.04E-17	2.04E-15
miR-493/136	DAVICIONI_MOLECULAR_ARMS_VS_ERMS_DN	182	15	0.0824	1.05E-17	2.04E-15
miR-493/136	WWTAAGGC_UNKNOWN	1896	34	0.0179	1.06E-17	2.04E-15
miR-493/136	GO_MULTICELLULAR_ORGANISMAL_MACROMOLECULE_METABOLIC_PROCESS	79	12	0.1519	1.08E-17	2.04E-15
miR-493/136	YAO_TEMPORAL_RESPONSE_TO_PROGESTERONE_CLUSTER_16	79	12	0.1519	1.08E-17	2.04E-15
miR-493/136	WESTON_VEGFA_TARGETS	108	13	0.1204	1.09E-17	2.04E-15
miR-493/136	CUI_TCF21_TARGETS_UP	37	10	0.2703	1.11E-17	2.05E-15
miR-493/136	MIYAGAWA_TARGETS_OF_EWSR1_ETS_FUSIONS_DN	229	16	0.0699	1.16E-17	2.13E-15
miR-493/136	REACTOME_COLLAGEN_FORMATION	58	11	0.1897	1.88E-17	3.41E-15
miR-493/136	RODWELL_AGING_KIDNEY_UP	487	20	0.0411	2.66E-17	4.78E-15
miR-493/136	GSE1460_CD4_THYMOCYTE_VS_THYMIC_STROMAL_CELL_DN	200	15	0.075	4.35E-17	7.73E-15
miR-379/656	SCHUETZ_BREAST_CANCER_DUCTAL_INVASIVE_UP	351	64	0.1823	3.58E-106	6.36E-102
miR-379/656	BOQUEST_STEM_CELL_UP	260	53	0.2038	2.40E-89	2.13E-85
miR-379/656	ANASTASSIOU_MULTICANCER_INVASIVENESS_SIGNATURE	64	39	0.6094	5.92E-88	3.51E-84
miR-379/656	GO_EXTRACELLULAR_MATRIX	426	54	0.1268	4.21E-79	1.87E-75
miR-379/656	HALLMARK_EPITHELIAL_MESENCHYMAL_TRANSITION	200	45	0.225	2.60E-77	9.26E-74
miR-379/656	MODULE_47	225	45	0.2	9.55E-75	2.83E-71
miR-379/656	GO_PROTEINACEOUS_EXTRACELLULAR_MATRIX	356	49	0.1376	3.91E-73	9.93E-70
miR-379/656	NABA_CORE_MATRISOME	275	46	0.1673	1.39E-72	3.10E-69
miR-379/656	NABA_MATRISOME	1028	58	0.0564	1.80E-64	3.56E-61
miR-379/656	GO_EXTRACELLULAR_STRUCTURE_ORGANIZATION	304	39	0.1283	2.62E-56	4.66E-53
miR-379/656	TURASHVILI_BREAST_LOBULAR_CARCINOMA_VS_DUCTAL_NORMAL_UP	69	26	0.3768	5.39E-51	8.71E-48
miR-379/656	TURASHVILI_BREAST_LOBULAR_CARCINOMA_VS_LOBULAR_NORMAL_DN	74	25	0.3378	1.37E-47	2.03E-44
miR-379/656	GO_EXTRACELLULAR_MATRIX_COMPONENT	125	28	0.224	1.62E-47	2.22E-44
miR-379/656	MODULE_1	368	35	0.0951	1.26E-45	1.60E-42
miR-379/656	ONDER_CDH1_TARGETS_2_UP	256	32	0.125	1.36E-45	1.62E-42
miR-379/656	FARMER_BREAST_CANCER_CLUSTER_4	19	17	0.8947	2.48E-43	2.76E-40
miR-379/656	LIM_MAMMARY_STEM_CELL_UP	489	35	0.0716	3.28E-41	3.43E-38
miR-379/656	NABA_ECM_GLYCOPROTEINS	196	26	0.1327	1.05E-37	1.04E-34
miR-379/656	MODULE_122	141	24	0.1702	1.35E-37	1.27E-34
miR-379/656	CHICAS_RB1_TARGETS_CONFLUENT	567	34	0.06	2.27E-37	2.02E-34
miR-379/656	LINDGREN_BLADDER_CANCER_CLUSTER_2B	392	30	0.0765	4.17E-36	3.53E-33
miR-379/656	VECCHI_GASTRIC_CANCER_ADVANCED_VS_EARLY_UP	175	24	0.1371	3.46E-35	2.79E-32
miR-379/656	PICCALUGA_ANGIOIMMUNOBLASTIC_LYMPHOMA_UP	205	24	0.1171	1.87E-33	1.45E-30
miR-379/656	GO_EXTRACELLULAR_SPACE	1376	41	0.0298	3.90E-33	2.89E-30
miR-379/656	SWEET_LUNG_CANCER_KRAS_DN	435	28	0.0644	1.47E-31	1.04E-28
miR-379/656	BERENJENO_TRANSFORMED_BY_RHOA_DN	394	27	0.0685	3.59E-31	2.45E-28
miR-379/656	CHARAFE_BREAST_CANCER_LUMINAL_VS_MESENCHYMAL_DN	460	28	0.0609	7.03E-31	4.63E-28
miR-379/656	KIM_GLIS2_TARGETS_UP	84	18	0.2143	2.57E-30	1.63E-27
miR-379/656	WEST_ADRENOCORTICAL_TUMOR_DN	546	28	0.0513	8.35E-29	5.12E-26
miR-379/656	WONG_ENDMETRIUM_CANCER_DN	82	17	0.2073	2.08E-28	1.23E-25
miR-379/656	MODULE_2	384	25	0.0651	2.58E-28	1.48E-25
miR-379/656	GNF2_CDH11	25	13	0.52	3.36E-28	1.87E-25
miR-379/656	CLASPER_LYMPHATIC_VESSELS_DURING_METASTASIS_DN	36	14	0.3889	5.29E-28	2.85E-25
miR-379/656	GO_EXTRACELLULAR_MATRIX_STRUCTURAL_CONSTITUENT	76	16	0.2105	6.56E-27	3.43E-24
miR-379/656	GNF2_PTX3	36	13	0.3611	1.46E-25	7.42E-23
miR-379/656	IGLESIAS_E2F_TARGETS_UP	151	18	0.1192	2.14E-25	1.06E-22
miR-379/656	WANG_SMARCE1_TARGETS_UP	280	21	0.075	3.67E-25	1.76E-22
miR-379/656	LIU_PROSTATE_CANCER_DN	481	24	0.0499	1.95E-24	9.12E-22
miR-379/656	MODULE_12	360	22	0.0611	2.30E-24	1.05E-21
miR-379/656	IZADPANAH_STEM_CELL_ADIPOSE_VS_BONE_DN	108	16	0.1481	2.89E-24	1.29E-21
miR-379/656	CUI_TCF21_TARGETS_2_UP	428	23	0.0537	3.61E-24	1.56E-21
miR-379/656	GO_COLLAGEN_TRIMER	88	15	0.1705	8.68E-24	3.68E-21
miR-379/656	PID_INTEGRIN1_PATHWAY	66	14	0.2121	1.01E-23	4.20E-21

miR-379/656	GO_BIOLOGICAL_ADHESION	1032	30	0.0291	1.10E-23	4.45E-21
miR-379/656	GO_BASEMENT_MEMBRANE	93	15	0.1613	2.12E-23	8.36E-21
miR-379/656	REN_ALVEOLAR_RHABDOMYOSARCOMA_DN	408	22	0.0539	3.57E-23	1.38E-20
miR-379/656	CHEN_METABOLIC_SYNDROM_NETWORK	1210	31	0.0256	7.19E-23	2.72E-20
miR-379/656	GO_MULTICELLULAR_ORGANISMAL_MACROMOLECULE_METABOLIC_PROCESS	79	14	0.1772	1.59E-22	5.88E-20
miR-379/656	BRUINS_UVC_RESPONSE_LATE	1137	30	0.0264	1.74E-22	6.30E-20
miR-379/656	REACTOME_COLLAGEN_FORMATION	58	13	0.2241	1.91E-22	6.78E-20
miR-379/656	NABA_COLLAGENS	44	12	0.2727	5.90E-22	2.02E-19
miR-379/656	TURASHVILI_BREAST_DUCTAL_CARCINOMA_VS_DUCTAL_NORMAL_UP	44	12	0.2727	5.90E-22	2.02E-19
miR-379/656	SENESE_HDAC1_AND_HDAC2_TARGETS_DN	232	18	0.0776	6.06E-22	2.03E-19
miR-379/656	REACTOME_EXTRACELLULAR_MATRIX_ORGANIZATION	87	14	0.1609	6.79E-22	2.24E-19
miR-379/656	RIGGI_EWING_SARCOMA_PROGENITOR_DN	191	17	0.089	8.33E-22	2.69E-19
miR-379/656	GO_COLLAGEN_BINDING	65	13	0.2	9.79E-22	3.11E-19
miR-379/656	PID_SYNDECAN_1_PATHWAY	46	12	0.2609	1.08E-21	3.38E-19
miR-379/656	GO_MULTICELLULAR_ORGANISM_METABOLIC_PROCESS	93	14	0.1505	1.84E-21	5.64E-19
miR-379/656	SERVITJA_ISLET_HNF1A_TARGETS_UP	163	16	0.0982	2.82E-21	8.50E-19
miR-379/656	LIEN_BREAST_CARCINOMA_METAPLASTIC	35	11	0.3143	5.29E-21	1.57E-18
miR-379/656	CHIANG_LIVER_CANCER_SUBCLASS_CTNNB1_DN	170	16	0.0941	5.63E-21	1.64E-18
miR-379/656	GO_REGULATION_OF_MULTICELLULAR_ORGANISMAL_DEVELOPMENT	1672	33	0.0197	6.92E-21	1.99E-18
miR-379/656	PID_AVB3_INTEGRIN_PATHWAY	75	13	0.1733	7.39E-21	2.09E-18
miR-379/656	CUI_TCF21_TARGETS_UP	37	11	0.2973	1.08E-20	3.00E-18
miR-379/656	RB_P107_DN.V1_UP	140	15	0.1071	1.35E-20	3.68E-18
miR-379/656	WESTON_VEGFA_TARGETS_6HR	59	12	0.2034	3.04E-20	8.18E-18
miR-379/656	DURAND_STROMA_S_UP	297	18	0.0606	5.28E-20	1.38E-17
miR-379/656	LEE_BMP2_TARGETS_UP	745	24	0.0322	5.28E-20	1.38E-17
miR-379/656	GO_ORGAN_MORPHOGENESIS	841	25	0.0297	5.48E-20	1.41E-17
miR-379/656	GSE1460_CD4_THYMOCYTE_VS_THYMIC_STROMAL_CELL_DN	200	16	0.08	7.98E-20	2.03E-17
miR-379/656	ZHU_CMV_24_HR_DN	91	13	0.1429	1.08E-19	2.71E-17
miR-379/656	ZHU_CMV_ALL_DN	128	14	0.1094	2.01E-19	4.96E-17
miR-379/656	JECHLINGER_EPITHELIAL_TO_MESENCHYMAL_TRANSITION_UP	71	12	0.169	3.39E-19	8.25E-17
miR-379/656	GO_VASCULATURE_DEVELOPMENT	469	20	0.0426	3.99E-19	9.59E-17
miR-379/656	MIYAGAWA_TARGETS_OF_EWSR1_ETS_FUSIONS_DN	229	16	0.0699	7.12E-19	1.69E-16
miR-379/656	WANG_MLL_TARGETS	289	17	0.0588	1.01E-18	2.36E-16
miR-379/656	YAO_TEMPORAL_RESPONSE_TO_PROGESTERONE_CLUSTER_16	79	12	0.1519	1.33E-18	3.08E-16
miR-379/656	SCHAEFFER_PROSTATE_DEVELOPMENT_48HR_DN	428	19	0.0444	1.56E-18	3.57E-16
miR-379/656	LANDIS_ERBB2_BREAST_TUMORS_324_DN	149	14	0.094	1.80E-18	4.05E-16
miR-379/656	CHICAS_RB1_TARGETS_GROWING	243	16	0.0658	1.85E-18	4.10E-16
miR-379/656	GO_COMPLEX_OF_COLLAGEN_TRIMERS	23	9	0.3913	2.04E-18	4.49E-16
miR-379/656	KEGG_ECM_RECEPTOR_INTERACTION	84	12	0.1429	2.91E-18	6.31E-16
miR-379/656	GO_CIRCULATORY_SYSTEM_DEVELOPMENT	788	23	0.0292	2.98E-18	6.38E-16
miR-379/656	GO_OSSIFICATION	251	16	0.0637	3.10E-18	6.56E-16
miR-379/656	GO_GLYCOSAMINOGLYCAN_BINDING	205	15	0.0732	4.64E-18	9.70E-16
miR-379/656	GO_SKELETAL_SYSTEM_DEVELOPMENT	455	19	0.0418	4.86E-18	1.00E-15
miR-379/656	CROMER_TUMORIGENESIS_UP	63	11	0.1746	7.37E-18	1.51E-15
miR-379/656	TTGCWCAAY_CEBPB_02	1972	32	0.0162	9.06E-18	1.83E-15
miR-379/656	BERTUCCI_MEDULLARY_VS_DUCTAL_BREAST_CANCER_DN	169	14	0.0828	1.09E-17	2.17E-15
miR-379/656	ESC_V6.5_UP_EARLY.V1_DN	172	14	0.0814	1.40E-17	2.76E-15
miR-379/656	RODWELL_AGING_KIDNEY_NO_BLOOD_UP	222	15	0.0676	1.54E-17	3.01E-15
miR-379/656	SENESE_HDAC2_TARGETS_DN	133	13	0.0977	1.85E-17	3.58E-15
miR-379/656	LANDIS_BREAST_CANCER_PROGRESSION_DN	70	11	0.1571	2.55E-17	4.88E-15
miR-379/656	THUM_MIR21_TARGETS_HEART_DISEASE_UP	17	8	0.4706	2.66E-17	5.00E-15
miR-379/656	WWTAAGGC_UNKNOWN	1896	31	0.0164	2.67E-17	5.00E-15
miR-379/656	GO_REGULATION_OF_CELLULAR_COMPONENT_MOVEMENT	771	22	0.0285	2.80E-17	5.18E-15
miR-379/656	HOSHIDA_LIVER_CANCER_SUBCLASS_S1	237	15	0.0633	4.11E-17	7.54E-15
miR-379/656	CHIARADONNA_NEOPLASTIC_TRANSFORMATION_KRAS_DN	142	13	0.0915	4.43E-17	8.04E-15
miR-379/656	GO_TISSUE_DEVELOPMENT	1518	28	0.0184	5.95E-17	1.07E-14
miR-379/656	LEE_NEURAL_CREST_STEM_CELL_UP	146	13	0.089	6.41E-17	1.14E-14
miR-199a/214	SCHUETZ_BREAST_CANCER_DUCTAL_INVASIVE_UP	351	59	0.1681	1.14E-99	2.03E-95
miR-199a/214	ANASTASSIOU_MULTICANCER_INVASIVENESS_SIGNATURE	64	38	0.5938	1.19E-87	1.05E-83
miR-199a/214	MODULE_47	225	46	0.2044	2.35E-80	1.39E-76
miR-199a/214	BOQUEST_STEM_CELL_UP	260	44	0.1692	1.07E-72	4.76E-69
miR-199a/214	HALLMARK_EPITHELIAL_MESENCHYMAL_TRANSITION	200	41	0.205	3.13E-71	1.11E-67
miR-199a/214	GO_EXTRACELLULAR_MATRIX	426	45	0.1056	1.19E-64	3.52E-61
miR-199a/214	NABA_MATRISOME	1028	52	0.0506	1.43E-58	3.62E-55
miR-199a/214	GO_PROTEINACEOUS_EXTRACELLULAR_MATRIX	356	40	0.1124	3.51E-58	7.80E-55
miR-199a/214	NABA_CORE_MATRISOME	275	37	0.1345	1.12E-56	2.21E-53
miR-199a/214	GO_EXTRACELLULAR_STRUCTURE_ORGANIZATION	304	34	0.1118	4.68E-49	8.32E-46
miR-199a/214	FARMER_BREAST_CANCER_CLUSTER_4	19	17	0.8947	2.22E-44	3.59E-41
miR-199a/214	TURASHVILI_BREAST_LOBULAR_CARCINOMA_VS_LOBULAR_NORMAL_DN	74	23	0.3108	2.87E-44	4.25E-41
miR-199a/214	MODULE_1	368	32	0.087	1.82E-42	2.48E-39
miR-199a/214	ONDER_CDH1_TARGETS_2_UP	256	29	0.1133	7.33E-42	9.31E-39
miR-199a/214	LINDGREN_BLADDER_CANCER_CLUSTER_2B	392	32	0.0816	1.45E-41	1.71E-38
miR-199a/214	TURASHVILI_BREAST_LOBULAR_CARCINOMA_VS_DUCTAL_NORMAL_UP	69	21	0.3043	3.22E-40	3.58E-37
miR-199a/214	LIM_MAMMARY_STEM_CELL_UP	489	33	0.0675	4.01E-40	4.19E-37
miR-199a/214	GO_EXTRACELLULAR_MATRIX_COMPONENT	125	23	0.184	2.42E-38	2.40E-35
miR-199a/214	CHICAS_RB1_TARGETS_CONFLUENT	567	33	0.0582	5.53E-38	5.17E-35
miR-199a/214	PICCALUGA_ANGIOIMMUNOBLASTIC_LYMPHOMA_UP	205	25	0.122	6.88E-37	6.11E-34
miR-199a/214	MODULE_122	141	22	0.156	5.54E-35	4.69E-32
miR-199a/214	LIU_PROSTATE_CANCER_DN	481	29	0.0603	1.01E-33	8.14E-31
miR-199a/214	CHARAFE_BREAST_CANCER_LUMINAL_VS_MESENCHYMAL_DN	460	28	0.0609	1.16E-32	8.97E-30
miR-199a/214	GO_EXTRACELLULAR_SPACE	1376	38	0.0276	7.23E-32	5.36E-29
miR-199a/214	VECCHI_GASTRIC_CANCER_ADVANCED_VS_EARLY_UP	175	20	0.1143	5.53E-29	3.79E-26
miR-199a/214	GNF2_CDH11	25	13	0.52	5.54E-29	3.79E-26
miR-199a/214	CHEN_METABOLIC_SYNDROM_NETWORK	1210	34	0.0281	1.38E-28	9.07E-26

miR-199a/214	REN_ALVEOLAR_RHABDOMYOSARCOMA_DN	408	24	0.0588	1.25E-27	7.97E-25
miR-199a/214	WEST_ADRENOCORTICAL_TUMOR_DN	546	26	0.0476	1.51E-27	9.26E-25
miR-199a/214	WANG_SMARCE1_TARGETS_UP	280	21	0.075	1.88E-26	1.11E-23
miR-199a/214	BERENJENO_TRANSFORMED_BY_RHOA_DN	394	23	0.0584	2.07E-26	1.19E-23
miR-199a/214	CLASPER_LYMPHATIC_VESSELS_DURING_METASTASIS_DN	36	13	0.3611	2.41E-26	1.34E-23
miR-199a/214	GO_EXTRACELLULAR_MATRIX_STRUCTURAL_CONSTITUENT	76	15	0.1974	9.85E-26	5.31E-23
miR-199a/214	CHIANG_LIVER_CANCER_SUBCLASS_CTNNB1_DN	170	18	0.1059	1.57E-25	8.23E-23
miR-199a/214	ESC_V6.5_UP_EARLY.V1_DN	172	18	0.1047	1.96E-25	9.93E-23
miR-199a/214	SWEET_LUNG_CANCER_KRAS_DN	435	23	0.0529	2.01E-25	9.93E-23
miR-199a/214	MODULE_2	384	22	0.0573	4.25E-25	2.04E-22
miR-199a/214	KIM_GLIS2_TARGETS_UP	84	15	0.1786	5.07E-25	2.37E-22
miR-199a/214	NABA_ECM_GLYCOPROTEINS	196	18	0.0918	2.22E-24	1.01E-21
miR-199a/214	SERVITJA_ISLET_HNF1A_TARGETS_UP	163	17	0.1043	4.87E-24	2.17E-21
miR-199a/214	GNF2_PTX3	36	12	0.3333	6.81E-24	2.95E-21
miR-199a/214	RODWELL_AGING_KIDNEY_NO_BLOOD_UP	222	18	0.0811	2.20E-23	9.32E-21
miR-199a/214	RB_P107_DN.V1_UP	140	16	0.1143	2.46E-23	1.02E-20
miR-199a/214	SENESE_HDAC1_AND_HDAC2_TARGETS_DN	232	18	0.0776	4.93E-23	1.99E-20
miR-199a/214	RIGGI_EWING_SARCOMA_PROGENITOR_DN	191	17	0.089	7.84E-23	3.10E-20
miR-199a/214	REACTOME_EXTRACELLULAR_MATRIX_ORGANIZATION	87	14	0.1609	9.80E-23	3.79E-20
miR-199a/214	TURASHVILI_BREAST_DUCTAL_CARCINOMA_VS_DUCTAL_NORMAL_UP	44	12	0.2727	1.13E-22	4.28E-20
miR-199a/214	GO_COLLAGEN_TRIMER	88	14	0.1591	1.16E-22	4.31E-20
miR-199a/214	IZADPANAH_STEM_CELL_ADIPOSE_VS_BONE_DN	108	14	0.1296	2.44E-21	8.86E-19
miR-199a/214	GO_MULTICELLULAR_ORGANISMAL_MACROMOLECULE_METABOLIC_PROCESS	79	13	0.1646	2.56E-21	9.09E-19
miR-199a/214	HOSHIDA_LIVER_CANCER_SUBCLASS_S1	237	17	0.0717	3.28E-21	1.14E-18
miR-199a/214	WONG_ENDMETRIUM_CANCER_DN	82	13	0.1585	4.30E-21	1.47E-18
miR-199a/214	REACTOME_COLLAGEN_FORMATION	58	12	0.2069	4.67E-21	1.57E-18
miR-199a/214	IGLESIAS_E2F_TARGETS_UP	151	15	0.0993	5.49E-21	1.81E-18
miR-199a/214	GO_BIOLOGICAL_ADHESION	1032	26	0.0252	1.45E-20	4.67E-18
miR-199a/214	ZHU_CMV_24_HR_DN	91	13	0.1429	1.82E-20	5.77E-18
miR-199a/214	GO_COLLAGEN_BINDING	65	12	0.1846	2.08E-20	6.49E-18
miR-199a/214	NABA_COLLAGENS	44	11	0.25	2.12E-20	6.50E-18
miR-199a/214	GO_BASEMENT_MEMBRANE	93	13	0.1398	2.45E-20	7.27E-18
miR-199a/214	GO_MULTICELLULAR_ORGANISM_METABOLIC_PROCESS	93	13	0.1398	2.45E-20	7.27E-18
miR-199a/214	PID_INTEGRIN1_PATHWAY	66	12	0.1818	2.54E-20	7.40E-18
miR-199a/214	ZHU_CMV_ALL_DN	128	14	0.1094	2.93E-20	8.41E-18
miR-199a/214	PID_SYNDECAN_1_PATHWAY	46	11	0.2391	3.67E-20	1.04E-17
miR-199a/214	SASAI_RESISTANCE_TO_NEOPLASTIC_TRANSFROMATION	50	11	0.22	1.02E-19	2.84E-17
miR-199a/214	PID_AVB3_INTEGRIN_PATHWAY	75	12	0.16	1.33E-19	3.63E-17
miR-199a/214	LANDIS_ERBB2_BREAST_TUMORS_324_DN	149	14	0.094	2.64E-19	7.11E-17
miR-199a/214	CUI_TCF21_TARGETS_UP	37	10	0.2703	4.97E-19	1.32E-16
miR-199a/214	GO_COMPLEX_OF_COLLAGEN_TRIMERS	23	9	0.3913	6.02E-19	1.57E-16
miR-199a/214	RODWELL_AGING_KIDNEY_UP	487	19	0.039	1.27E-18	3.28E-16
miR-199a/214	CUI_TCF21_TARGETS_2_UP	428	18	0.0421	2.97E-18	7.53E-16
miR-199a/214	MIYAGAWA_TARGETS_OF_EWSR1_ETS_FUSIONS_DN	229	15	0.0655	3.18E-18	7.96E-16
miR-199a/214	MODULE_12	360	17	0.0472	3.98E-18	9.82E-16
miR-199a/214	DURAND_STROMA_S_UP	297	16	0.0539	5.10E-18	1.24E-15
miR-199a/214	GO_PROTEIN_COMPLEX_BINDING	935	23	0.0246	5.40E-18	1.30E-15
miR-199a/214	LANDIS_BREAST_CANCER_PROGRESSION_DN	70	11	0.1571	5.71E-18	1.35E-15
miR-199a/214	SENESE_HDAC1_TARGETS_DN	260	15	0.0577	2.14E-17	5.01E-15
miR-199a/214	YAO_TEMPORAL_RESPONSE_TO_PROGESTERONE_CLUSTER_16	79	11	0.1392	2.35E-17	5.42E-15
miR-199a/214	BRUINS_UVC_RESPONSE_LATE	1137	24	0.0211	2.91E-17	6.64E-15
miR-199a/214	PETRETTO_CARDIAC_HYPERTROPHY	34	9	0.2647	3.79E-17	8.53E-15
miR-199a/214	LIEN_BREAST_CARCINOMA_METAPLASTIC	35	9	0.2571	5.09E-17	1.13E-14
miR-199a/214	WESTON_VEGFA_TARGETS_6HR	59	10	0.1695	8.62E-17	1.89E-14
miR-199a/214	MODULE_5	434	17	0.0392	8.98E-17	1.95E-14
miR-199a/214	GRUETZMANN_PANCREATIC_CANCER_UP	358	16	0.0447	9.79E-17	2.10E-14
miR-199a/214	WANG_MLL_TARGETS	289	15	0.0519	1.04E-16	2.18E-14
miR-199a/214	WONG_ADULT_TISSUE_STEM_MODULE	721	20	0.0277	1.04E-16	2.18E-14
miR-199a/214	GO_REGULATION_OF_MULTICELLULAR_ORGANISMAL_DEVELOPMENT	1672	27	0.0161	1.68E-16	3.47E-14
miR-199a/214	CROMER_TUMORIGENESIS_UP	63	10	0.1587	1.74E-16	3.55E-14
miR-199a/214	SENESE_HDAC2_TARGETS_DN	133	12	0.0902	1.76E-16	3.55E-14
miR-199a/214	BLALOCK_ALZHEIMERS_DISEASE_UP	1691	27	0.016	2.22E-16	4.43E-14
miR-199a/214	CHICAS_RB1_TARGETS_GROWING	243	14	0.0576	2.70E-16	5.34E-14
miR-199a/214	GO_VASCULATURE_DEVELOPMENT	469	17	0.0362	3.24E-16	6.33E-14
miR-199a/214	WWTAAGGC_UNKNOWN	1896	28	0.0148	3.96E-16	7.66E-14
miR-199a/214	GO_CIRCULATORY_SYSTEM_DEVELOPMENT	788	20	0.0254	5.65E-16	1.08E-13
miR-199a/214	GU_PDEF_TARGETS_UP	71	10	0.1408	6.20E-16	1.16E-13
miR-199a/214	JECHLINGER_EPITHELIAL_TO_MESENCHYMAL_TRANSITION_UP	71	10	0.1408	6.20E-16	1.16E-13
miR-199a/214	GSE1460_CD4_THYMOCYTE_VS_THYMIC_STROMAL_CELL_DN	200	13	0.065	6.91E-16	1.28E-13
miR-199a/214	GO_ENDOPLASMIC_RETICULUM_LUMEN	201	13	0.0647	7.37E-16	1.35E-13
miR-199a/214	GO_NEGATIVE_REGULATION_OF_RESPONSE_TO_STIMULUS	1360	24	0.0176	1.55E-15	2.81E-13
miR-199a/214	LINDVALL_IMMORTALIZED_BY_TERT_DN	80	10	0.125	2.17E-15	3.91E-13
miR-199a/214	GO_MACROMOLECULAR_COMPLEX_BINDING	1399	24	0.0172	2.89E-15	5.13E-13
